# Rapid *zero-trans* kinetics of Cs^+^ exchange in human erythrocytes quantified by dissolution hyperpolarized ^133^Cs^+^ NMR spectroscopy

**DOI:** 10.1038/s41598-019-56250-z

**Published:** 2019-12-23

**Authors:** Philip W. Kuchel, Magnus Karlsson, Mathilde Hauge Lerche, Dmitry Shishmarev, Jan Henrik Ardenkjaer-Larsen

**Affiliations:** 1 The University of Sydney, School of Life and Environmental Sciences, Sydney, New South Wales Australia; 2 Center for Hyperpolarization in Magnetic Resonance, Department of Health Technology, Technical University of Denmark, Lyngby, Denmark; 3 The Australian National University, John Curtin School of Medical Research, Canberra, Australian Capital Territory Australia

**Keywords:** Biophysics, Biotechnology

## Abstract

Transmembrane flux of Cs^+^ (a K^+^ congener) was measured in human red blood cells (RBCs; erythrocytes) on the 10-s time scale. This is the first report on dissolution dynamic nuclear polarization (dDNP) nuclear magnetic resonance (NMR) spectroscopy with this nuclide in mammalian cells. Four technical developments regularized sample delivery and led to high quality NMR spectra. Cation-free media with the Piezo1 (mechanosensitive cation channel) activator yoda1 maximized the extent of membrane transport. First-order rate constants describing the fluxes were estimated using a combination of statistical methods in *Mathematica*, including the Markov chain Monte Carlo (MCMC) algorithm. Fluxes were in the range 4–70 μmol Cs^+^ (L RBC)^−1^ s^−1^; these are smaller than for urea, but comparable to glucose. Methodology and analytical procedures developed will be applicable to transmembrane cation transport studies in the presence of additional Piezo1 effectors, to other cellular systems, and potentially *in vivo*.

## Introduction

### Motivation

Our aim was to use the newly available ability to measure the early stages (seconds) of cation transport in cells; and to determine conformity (or not) of these rates with later stages (minutes). This would be exploring whether a phenomenon like ‘hysteretic enzyme’ kinetics^1^ exists with the transport protein(s). And, to relate these fluxes to the time scales (kinetics) of other solutes that undergo transport into and out of cells across their plasma membranes. Thus, we would inform designs for *in vivo* and clinical applications of dissolution dynamic nuclear polarization (dDNP) nuclear magnetic resonance (NMR) spectroscopy with ^133^Cs^+^.

### Caesium NMR

^133^Cs^+^ is a congener of K^+^ that has much more favourable NMR properties (100% natural abundance, higher natural relative receptivity by a factor of 102, larger chemical shift range, and narrower linewidth, despite having a spin-quantum number of 7/2) than the magnetic ^39^K^+^ (spin quantum number 3/2)^2^. Moreover, ^133^Cs^+^ displays the remarkable feature that solutions of the ion inside and outside cells in suspensions (and tissues) give separate NMR peaks, amounting to ~4 ppm separation in the present studies^3,4^. This phenomenon, referred to as the “split-peak effect”^5^ arises in the absence of added NMR-shift reagents that could potentially interfer with proteins that mediate the transmembrane exchange of Cs^+^ and other cations.

### NMR signal enhancement

dDNP is a recently developed technique that increases the sensitivity for detection of many NMR-receptive nuclides by factors of ~10^4^ ^6^. In the context of ^133^Cs^+^, this opened up the possibility of recording transmembrane flux of the cation into cells^7^ on a timescale of several seconds and thus determine kinetic rate constants that can provide mechanistic insights into its membrane-transport processes.

### Red blood cell cation transport

We studied freshly drawn, metabolically active, human erythrocytes (red blood cells; RBCs) that were suspended in isotonic sucrose solution (300 mM), with glucose (15 mM) as the metabolic substrate. The lack of other cations avoided their competition with ^133^Cs^+^, thus enhancing uptake. Addition of the compound yoda1, an activator of the mechanosensitive non-selective cation channel Piezo1, further enhanced the rate of ^133^Cs^+^ membrane transport. ^133^Cs NMR spectra were recorded every 1 s and they produced time courses that were analyzed by fitting a model based on the numerical solution of the differential equations that described the kinetics of magnetization-relaxation and chemical exchange. Statistically robust estimates of the rate constants for hyperpolarized ^133^Cs^+^ transport were obtained for a range of initial ^133^Cs^+^ concentrations in *zero trans* experiments in which the ‘extent of reaction’ was routinely <1%. By ‘*zero trans*’ we mean that the concentration of the transported solute (in this case ^133^Cs^+^) is initially zero on the opposite (*trans*) side of the membrane^8–10^. The estimated rate constants closely matched those from much longer time courses recorded using conventional NMR acquisition (a spectrum every 10–20 s) of non-hyperpolarized ^133^Cs NMR spectra over a total period of 20 min.

### Technical developments

Four technical and experimental extensions were implemented to improve previous dDNP sample configurations in order to obtain reproducible kinetic data on ^133^Cs^+^ uptake into RBCs on the ~10 second time scale. In particular, we removed irregularities in NMR lineshape that might arise from incomplete mixing of the dDNP solution, thermal gradients, and the presence of a sample-delivery tube in the RBC suspension, as follows: (1) The hyperpolarized ^133^Cs^+^ with its dissolution medium was thermally equilibrated at 37 °C during the process of its injection into the RBC suspension. This occurred via a custom-built counter-current heat-exchanger that was an adaptation of an earlier model^11^ that now has four delivery tubes instead of one through the axial bore (one for the solution and two for air actuation of a piston, see below; plus a spare one). Small o-rings sealed each of the tubes at both the top and bottom of the device (Technical drawing in Fig. S1, Supplementary Information).

A syringe-based delivery system for the dDNP solutions was designed and implemented. It achieved: (2) uniform delivery and mixing by what is conceptually a layering of the added solution into the RBC suspension by, (3) simultaneously withdrawing the delivery tube from within the RBC suspension and out of the active volume of the NMR receiver coil. (Supplementary Information has a full description of the device and its operation.) (4) In preliminary experiments with RBCs suspended in physiological saline with glucose (154 mM NaCl, 15 mM D-glucose), little uptake of hyperpolarized ^133^Cs^+^ was evident from the ^133^Cs NMR spectra. This was ascribed to competition for transport, via Piezo1 (a non-selective cation channel), by the very abundant Na^+^ in the saline solution. Therefore, the medium was changed to isotonic sucrose containing glucose as the energy source (300 mM sucrose, 15 mM glucose), similar to what had been used in related experiments using thermal NMR^12^.

## Results

### Validation of injection system

**Figure 1:**
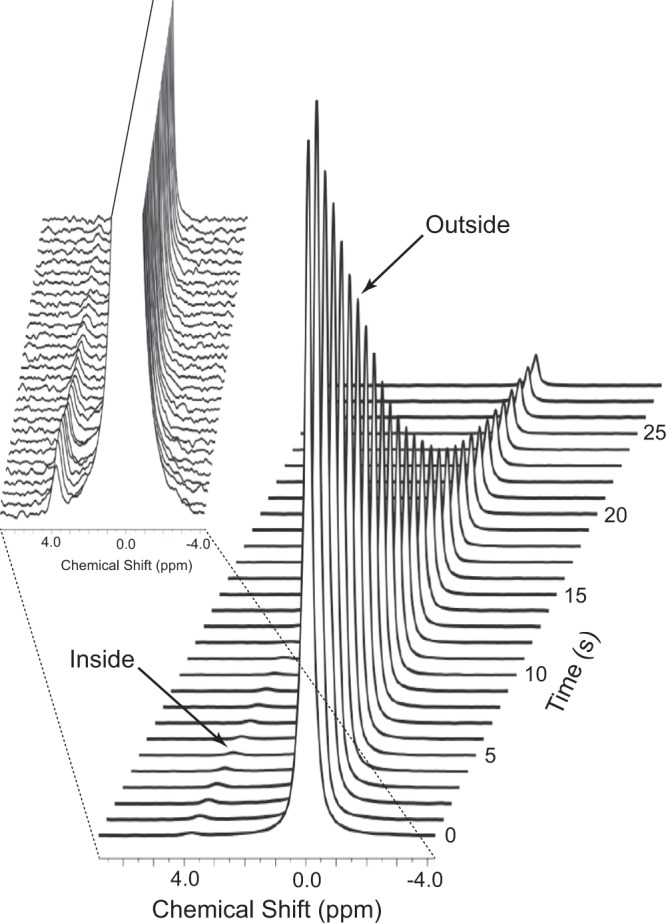
^133^Cs-NMR (52.5 MHz) spectral time course of hyperpolarized ^133^Cs^+^ (initially 3.3 mM) entering human RBCs treated with yoda1, at 37 °C. Sample: 1.5 mL of RBCs suspended in 300 mM sucrose and 15 mM glucose treated with yoda1 [80 μL of 14 mM stock solution per 2.5 mL of haematocrit (*Ht)* = 0.8 in 15 mL total volume; implying a solution concentration of 75 μM, or in terms of the RBCs present in the suspension, 37 mmol (L RBC)^−1^], injected with 1.5 mL of solution containing hyperpolarized ^133^Cs^+^ at the initial extracellular concentration of 3.3 mM. NMR: Spectra were recorded every 1 s, using a notional RF magnetization-sampling pulse-angle of 10°; free induction decays (FIDs) consisted of 8 K complex data points; these were processed with a decaying exponential line-broadening factor of 5 Hz; the chemical shift scale was arbitrarily set to 0.000 ppm on the peak from extracellular ^133^Cs^+^, making the peak from intracellular ^133^Cs^+^ to be centred at ~4 ppm. In the first spectrum, the amplitude of the peak from extracellular ^133^Cs^+^ was smaller than in the second spectrum, but its width-at-half-height was larger than in subsequent spectra and its overall intensity (area) was the greatest. The inset shows the stack plot with much larger vertical scale to accentuate the small, emerging, and then declining, peak from intracellular ^133^Cs^+^.

Figure 1 shows a time course of ^133^Cs NMR spectra recorded every 1 s after the rapid injection of a solution of hyperpolarized ^133^Cs^+^ into a suspension of metabolically active RBCs, at 37 °C. The peak from the extracellular ^133^Cs^+^ is clearly the dominant feature, and its uniform Lorentzian line-shape is evident. The intracellular resonance at ~4 ppm to higher frequency of the extracellular one emerged rapidly and decayed into the baseline over ~40 s. This provided sufficiently reliable data to allow statistically robust estimates of the values of the longitudinal relaxation time constants, *T*_1,i_ and *T*_1,o_ (i and o denote inside and outside), and the influx rate constant *k*_1_ (see more details on the data analysis below).

### Relaxation and kinetic rate constants

**Figure 2:**
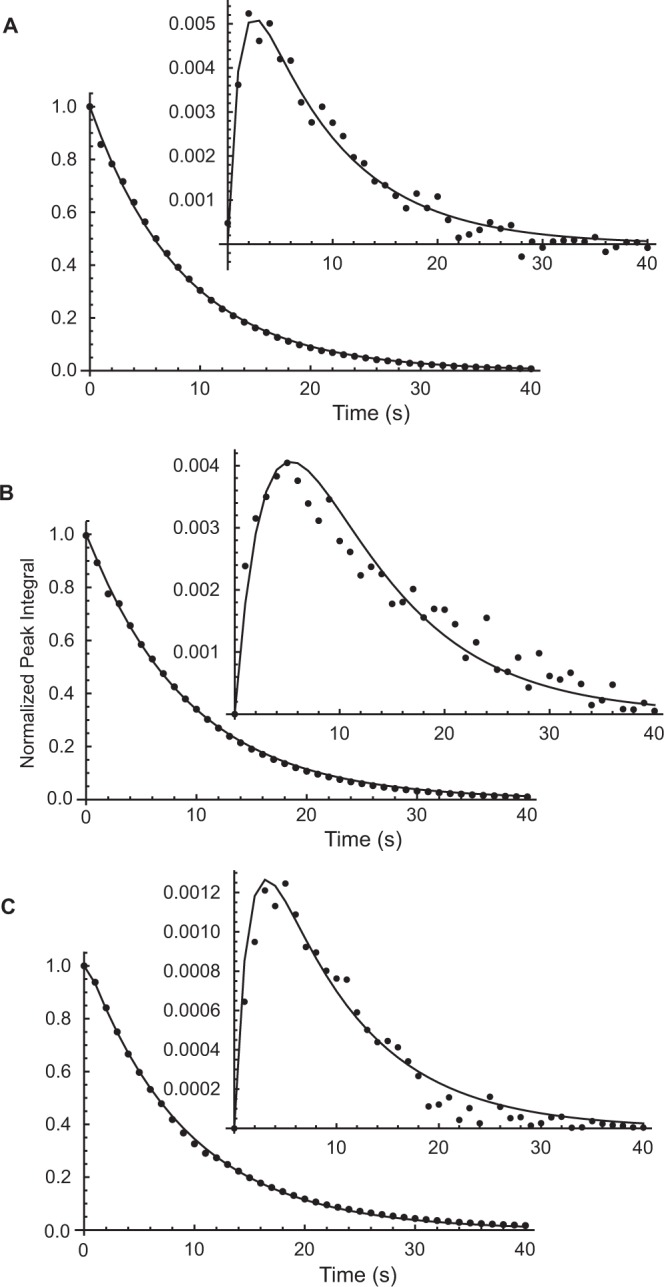
Time courses of ^133^Cs NMR (52.5 MHz) peak integrals from hyperpolarized ^133^Cs^+^ in suspensions of human RBCs treated with yoda1, at 37 °C. Dots are the experimental data points while the solid lines are fits of the solutions of the differential equations that describe a two-site exchange process (Materials and Methods) by using an MCMC algorithm in *Mathematica*^32,34^. Samples: (**A**) same as for Fig. 1, *viz*., 3.3 mM hyperpolarized ^133^CsCl in 300 mM sucrose containing 15 mM D-glucose in RBCs treated with 80 μL of 14 mM yoda1 in DMSO in the sample preparation stage, and 1.5 mL of these RBCs used for the time course. (**B**) 2.5 mM hyperpolarized ^133^CsCl, similar to (**A**) but with only 40 μL of 14 mM yoda1 in DMSO used in the RBC preparation stage, and with the RBCs washed in CsCl (33 mM), sucrose (222 mM) and D-glucose (11.1 mM); thus, the RBCs had become equilibrated with ‘unlabelled’ ^133^Cs^+^ beforehand. (**C**) 33.3 mM hyperpolarized ^133^CsCl in 300 mM sucrose containing 15 mM D-glucose in RBCs treated as described in Materials and Methods with 40 μL of 14 mM yoda1 in DMSO used in the RBC preparation stage. The estimates of *T*_1,o_ (s), *T*_1,i_ (s) and *k*_1_ (s^−1^) were, respectively: (**A**) 8.9 ± 0.1, 1.2 ± 0.1, 0.0057 ± 0.0006 (Table S2 Row 10); (**B**) 9.6 ± 0.2, 3.0 ± 0.7, 0.0024 ± 0.0006 (Table S2 Row 15); (**C**) 13.6 ± 1.5, 2.4 ± 0.6, 0.0008 ± 0.0002 (Table S2 Row 13). NMR: an FID was recorded every 1 s as 8 K complex data points, using a notional nutation RF pulse of 10°. The FID was multiplied with a decaying exponential function with a broadening factor of 5 Hz. Peak integrals were measured using MNova software (Materials and Methods).

Figure 2 shows three representative NMR peak intensity (integral) time courses from ^133^Cs^+^-dDNP experiments on identically composed samples of RBCs, but with different amounts of the Piezo1 activator, yoda1. The first two experiments had similar ^133^Cs^+^ concentration (3.3 mM and 2.5 mM, respectively) while the third had a much higher value (33.3 mM). It is evident in the latter series of spectra that, relative to the intensity of the extracellular peak, the intracellular peak was much smaller; specifically, the maximum relative intensity was only ~0.1% of the extracellular one. On the other hand, with the lower concentrations this value was ~0.5%. Overall, it is clear that the extent of reaction is tiny relative to what might be recorded in a conventional ‘thermal’ experiment (see below), so the dDNP measurements of influx constituted what is referred to in enzyme kinetics parlance as ‘initial velocity’ measurements.

### Variations of initial yoda1 and ^133^Cs^+^ concentrations

It became clear from experiments like those shown in Fig. 2 that in the dDNP experiments only tiny ‘extents of reaction’ were being recorded. Thus, the build-up curves for the intracellular ^133^Cs^+^ yielded estimates of true initial velocities. Accordingly, a simple model of the exchange process, which did not include the back-reaction (*k*_−1_) appeared justified for fitting to the data. (The implications of this simplification for describing longer time courses is taken up in the Discussion). This reduced the number of degrees of freedom in the regression analysis and improved the quality of fits of the model onto the data.

In the process of regression analysis, we also sought independent estimates of the longitudinal relaxation time constants of extra- and intracellular ^133^Cs^+^. The *T*_1_ values reported in Table S2, for a set of experiments recorded from the same batch of RBCs, consistently showed a six-fold greater value outside (9.6 ± 0.2 s) than inside (1.6 ± 0.1 s) the RBCs. On the other hand, the *k*_1_ values ranged over a factor of ~20 for the different experimental conditions from the lowest yoda1 concentration and highest initial [^133^Cs^+^] (Row 1) to the highest yoda1 concentration and lowest initial [^133^Cs^+^] (Row 12).

It may seem paradoxical that the highest initial [^133^Cs^+^] (Row 1) should yield the lowest estimate of *k*_1_; but this is the consequence of the lowest ‘extent of reaction’ in the whole data set whereas the flux, which is calculated from the product of *k*_1_ and the initial concentration was, as might be predicted, amongst the highest. (Whether the value of the flux is as high as expected for this Piezo1-mediated reaction is discussed further below.)

Comparing Rows 6–8 with Rows 9–12 in Table S2 showed that the change in yoda1 concentration did not have significant effect on the estimates of *k*_1_, based (simply) on a consideration of ‘overlap’ of the means by two-standard deviations. Therefore, we concluded that the concentration of yoda1 to be used [as noted above: 40 or 80 μL of 14 mM in dimethylsulfoxide (DMSO), added to 2.5 mL of RBCs of *Ht* = 0.8 in a total volume of 15 mL implying a concentration of 37 or 75 μM, respectively, averaged over the whole sample volume] was optimal for eliciting a saturated effect of yoda1 in these experiments. The DMSO used as the solvent for the yoda1 caused minimal haemolysis during the preparation of the RBCs for the experiment; this was an outcome that was consistent with earlier studies on the effects of organic solvents on RBCs^13,14^. Furthermore, in control experiments, DMSO used alone caused no enhancement of ^133^Cs^+^ entry, on the timescale of the dDNP experiments.

### ‘Thermal NMR’ time course analysis

#### Influx rate

**Figure 3:**
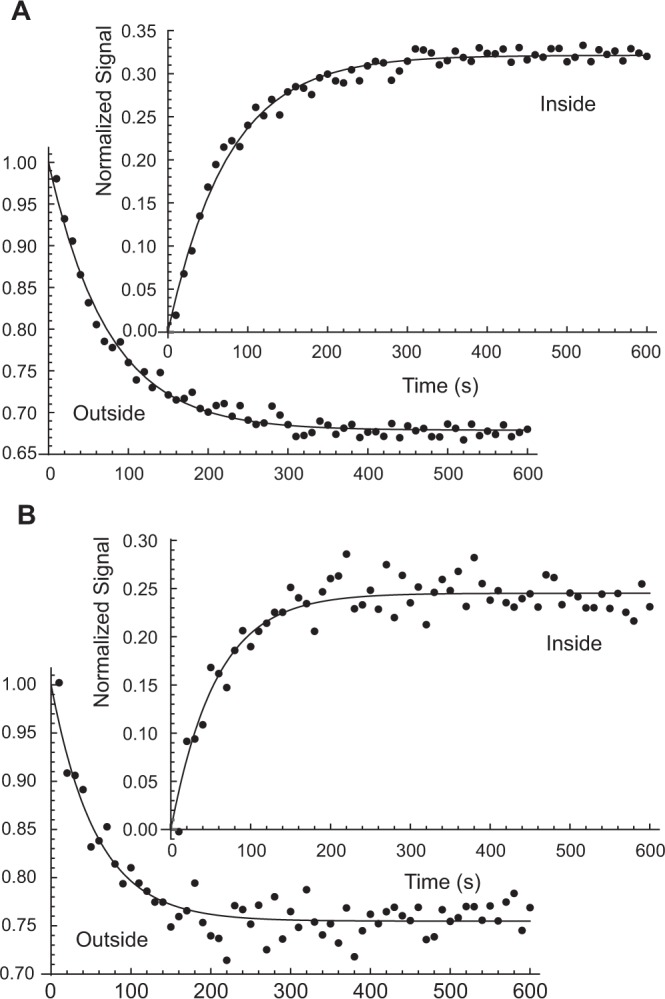
^133^Cs-NMR (52.5 MHz) peak integrals from two time-courses conducted at 37 °C, commencing with 33 mM CsCl outside that RBCs that had been treated with yoda1. [NB, The sum of the two peak integrals was used to normalize the relative amounts of ^133^Cs^+^ in each of the two compartments (conservation of mass condition), so the points in the progress curves are located at values that are mirror images of each other, around a virtual horizontal ordinate line at 0.5]. (**A**) Data were recorded after using the ‘D4 delivery system’ (Supplementary Information) and (**B)** was recorded after the direct delivery of the RBC suspension into the Cs^+^ solution via a tube that was then rapidly withdrawn from the sample. Black dots in the respective figures denote ^133^Cs^+^ outside and inside the RBCs. The solid lines are fits of the solutions of the differential equations that describe a two-site exchange reaction (Materials and Methods) by using the ParametricNDSolveValue and NonlinearModelFit functions in *Mathematica*^32,34^. (**A**) The first order rate constant estimated for the forward transport reaction *k*_1_ = 0.0043 ± 0.0001 s^−1^ and for the reverse reaction *k*_−1_ = 0.0091 ± 0.0002 s^−1^ (Table S1 Row 2). Sample: 1.5 mL of RBCs of *Ht* = 0.8 in 300 mM sucrose - 15 mM D-glucose, aliquoted from a 2.5 mL pellet that had been treated with 80 μL of 14 mM yoda1 in DMSO, as described in Materials and Methods. At *t* = 0 the RBC suspension was diluted with 1.5 mL of 40 mM CsCl in the sucrose-D-glucose solution; this CsCl solution was diluted to 33 mM by the interstitial medium [(1-*Ht*) × 1.5 mL = 0.3 mL, making the extracellular aqueous volume 1.8 mL] in the RBC suspension. (**B**) The value of the first order rate constant estimated for the forward reaction was *k*_1_ = 0.0043 ± 0.0002 and for the reverse reaction *k*_−1_ = 0.013 ± 0.0007 s^−1^ (Table S1 Row 6). Sample: 1.5 mL of RBCs of *Ht* = 0.8 in 300 mM sucrose - 15 mM D-glucose, aliquoted from a 2.5 mL pellet that had been treated with 40 μL of 14 mM yoda1 in DMSO, as described in Materials and Methods. At *t* = 0 the RBC suspension was diluted with 1.5 mL of 40 mM CsCl in the sucrose-D-glucose solution; this CsCl solution was diluted to 33 mM by the interstitial medium [(1 − *Ht*) × 1.5 mL = 0.3 mL, making the extracellular aqueous volume 1.8 mL] in the RBC suspension. NMR parameters: 10 complex FIDs of 8 K each were summed per spectrum, over 20 s, using an RF-pulse nutation angle of 30° that was based on the ‘Ernst angle’ calculation for a *T*_1_ of 10 s. The sum of the intra- and extra-cellular peak integrals was used to normalize the values for each pair of peaks to a total of 1.0.

Figure 3A shows a time course of peak integrals in a series of thermal NMR spectra of the suspension of RBCs (*Ht* = 0.8), which had previously been treated with yoda1, and then injected with ^133^Cs^+^ solution at an initial concentration of 16.7 mM. The peak amplitudes of the extra- and intra-cellular ^133^Cs^+^ ions declined, and rose, in seemingly mono-exponential manners (Fig. 3). The cation had equilibrated across the RBC membranes by ~10 min. ParametricNDSolveValue regression (*Mathematica* script in Supplementary Information) of the solutions of the two-site exchange kinetic equation (Methods Eqs. 4 and 5) gave estimates of the rate constants *k*_1_ and *k*_−1_ of (4.3 ± 0.1) × 10^−3^ s^−1^ and (9.1 ± 0.2) × 10^−3^ s^−1^, respectively. This corresponded to an initial flux of 0.0043 s^−1^ × 16.7 mM × (1/(*Ht* = 0.4)) = 0.18 mmol (L RBC)^−1^ s^−1^.

For Fig. 3B, a thermally equilibrated RBC suspension was injected into the ^133^Cs^+^ solution (that was already in the bore of the magnet) via a polyether ether ketone (PEEK) tube that was then rapidly withdrawn. The value of *k*_1_ estimated from the data was the same as for Fig. 3A, while the values of *k*_−1_ differed (see Table S1); this matter is discussed below.

#### Permeability coefficient

The diffusional permeability coefficient of a cell membrane is defined as^15^:

1 $${P}_{d}={k}_{-1}\frac{{V}_{{\rm{RBC}}}}{{A}_{{\rm{RBC}}}},$$

where *V*_RBC_ is the water volume of one RBC and *A*_RBC_ is its surface area, which for normal human RBCs are 62 fL and 143 μm^2^, respectively^16^. Hence, for the data in Fig. 3A, the permeability coefficient for Cs^+^ was, using the estimate of *k*_−1_ from the fit, *P*_Cs_ = 0.0091 s^−1^ × 62 fL/143 μm^2^ = 3.9 × 10^−7^ cm s^−1^. This is approximately an order of magnitude greater than the RBC’s permeability coefficient for Cl^−^ ^17^.

#### Membrane potential

The *Ht* of the RBC suspension became 0.4 upon dilution by the Cs^+^ solution. Hence the fraction of the sample that was intracellular water was 0.4 × α, where α is the volume fraction of the intracellular space of a normo-volumic human RBC, *viz*., 0.717^18^. This computes to 0.287, while the extracellular volume fraction (1 − *Ht*) was 0.6, thus giving the ratio of the aqueous volumes of 68:32, This ratio is exactly the same as the ratio of the extra- to intra-cellular signal at equilibrium in Fig. 3A. The implication of this finding is that the electrochemical equilibrium of the system displays the same *concentration* of ^133^Cs^+^ outside and inside the cells. On the other hand, under the different conditions used to acquire the data in Fig. 3B the concentration ratio was 1.19:1. If we apply the reasonable assumption that ion permeability is dominated typically by Cl^−^ (via capnophorin) and now additionally via Piezo1 enhanced by yoda1, the Goldman equation^9,19–21^ for the trans-membrane potential (Δ*ψ*) becomes:

2 $$\Delta \psi =\frac{R\,T}{z\,F}\,Ln\frac{{P}_{Cs}{[C{s}^{+}]}_{out}+{P}_{Cl}{[C{l}^{-}]}_{in}\,}{{P}_{Cs}{[C{s}^{+}]}_{in}+{P}_{Cl}{[C{l}^{-}]}_{out}}$$

where *R* (8.314 J mol^−1^ K^−1^) is the Universal gas constant, *T* is the absolute temperature (310.15 K), *z* is 1 for a monovalent positive ion, and *F* is the Faraday’s constant (96.5 kC mol^−1^). However, using Eq. 2, and knowing that the Cl^−^ permeability is *actually* significantly less than that of Cs^+^, the expression for the membrane potential simplifies to the Nernst equation:

3 $$\varDelta {\psi }_{Cs}=\frac{R\,T}{z\,F}\,Ln\frac{{[C{s}^{+}]}_{out}}{{[C{s}^{+}]}_{in}}$$

Equation 3 yielded the value of the transmembrane potential $\varDelta {\psi }_{Cs}=+\,4.6\,{\rm{mV}}$ for the data in Fig. 3B, which is of opposite sign to the typically reported one of ~−10 mV^18^. Four other experiments were performed and the results are given in Table S1 in the Supplementary Information.

## Discussion

### Experimental requirements and precursor studies

It was evident in initial experiments with ^133^Cs dDNP, that under conventional incubation conditions the rate of entry of ^133^Cs^+^ into human RBCs would not be of the same order of magnitude as seen with electroporated yeast cells^7^. However, based on experiments with RBCs that had been treated with yoda1, suspended in a sucrose-based medium (with 30 mM ^133^Cs^+^), the rate of entry would be sufficient for detection under *zero-trans* conditions^8–10^. This proved to be the case as exemplified by the spectral time course in Fig. 1 and the peak integral plots in Fig. 2. The influx of ^133^Cs^+^ at 37 °C in yoda1-treated RBCs, shown in Fig. S3 of our previous work^12^ was 1.74 mmol (L RBC)^−1^ min^−1^ or 29 μmol (L RBC)^−1^ s^−1^. This value is precisely in the range of those listed in the last column of Table S2.

### Comparisons with other solutes

3-fluoro-3-deoxy-D-glucose has an efflux rate constant in human RBCs of ~1 s^−1^ at 37 °C. This corresponds to a flux under normal physiological plasma concentrations (~5 mM) of ~30 μmoles (L RBC)^−1^ s^−1^ ^22,23^. This is very similar to the fluxes shown in the last column of Table S2, for Cs^+^ influx.

On the other hand, urea transmembrane exchange, either under equilibrium exchange^24^, or *zero-trans*^11^, conditions is substantially faster than yoda1-enhanced ^133^Cs^+^ influx, being ~50 mmol (L RBC) s^−1^ at a typical plasma concentration of ~5 mM. In other words, it is ~1000 times faster than the yoda1-enhanced ^133^Cs^+^ exchange. This is the primary reason its kinetics could be so readily characterized with ^13^C-dDNP^11^. In other words, the extent of the transport reaction involving hyperpolarized urea is typically high (Fig. 5 in^11^) compared with experiments with hyperpolarized ^133^Cs^+^.

### Extracting total flux values from dDNP data

**Figure 4:**
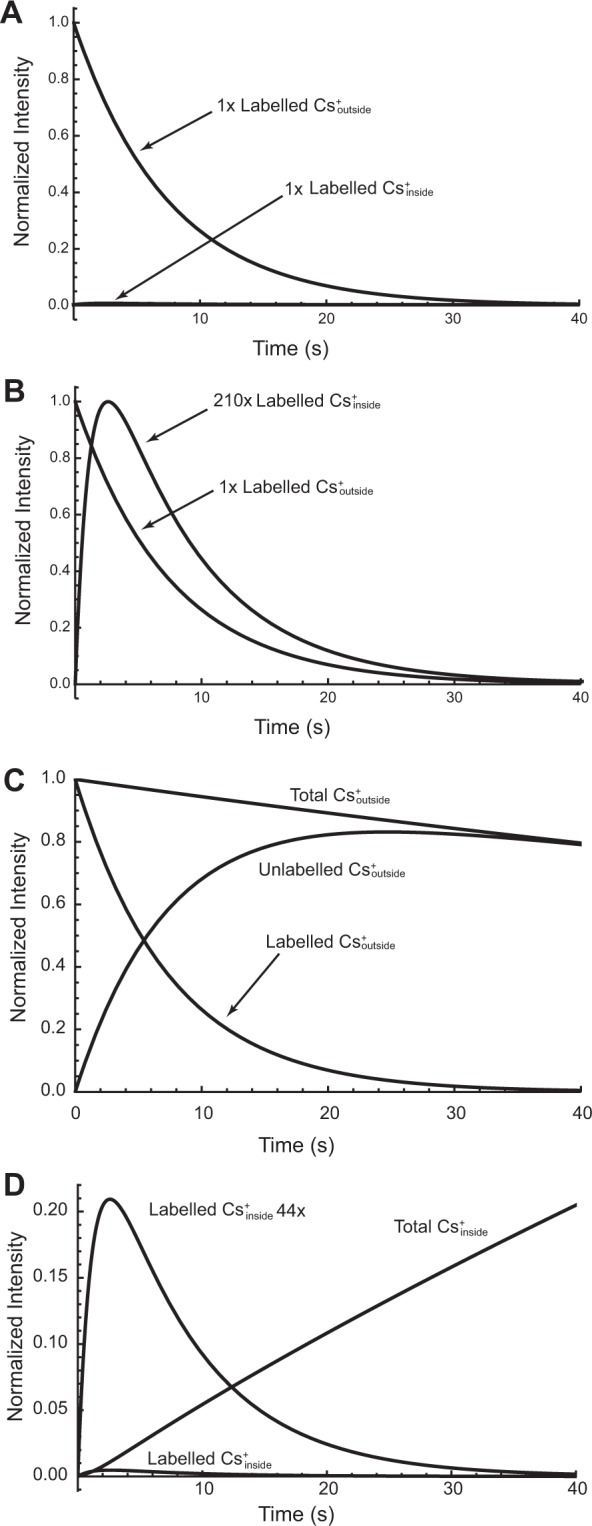
Simulations of ^133^Cs-NMR time-courses using the parameter values (*T*_1,o_, *T*_1,i_, *k*_1_, *k*_−1_, flip angle α of Fig. 2A and Table S2 Row 10; *viz*., 8.9 s, 1.2 s, 0.0057 s^−1^, 0.0 or 0.0121 s^−1^, and 10°) with numerical integration of Eqs. 4–7 using NDSolve in *Mathematica*^32^. (**A**) Simulated times courses of normalized dDNP signal intensity like those shown in Fig. 2A; (**B**) scaling of the intracellular signal to make the maximum of the curve have the value of 1.0; note the 210-fold scaling required to achieve this outcome. (**C**) Illustration of the net (Total) *chemical* flux that was calculated from the label flux. Note the flux of the unlabelled ^133^Cs^+^ that is the complement of the labelled ^133^Cs^+^. (**D**) Time course of the intracellular accumulation of ^133^Cs^+^ showing the tiny labelled pool relative to the unlabelled pool. The total pool mirrors that of the decline of the total extracellular pool shown in (**C**).

Figure 4 shows the results of numerical integration of Eqs. 4–7 using the values of the two longitudinal relaxation times, the influx rate constant and flip angle of the radiofrequency (RF) magnetization-sampling pulse, from the experiment shown in Figs. 1 and 2A. Figure 4A underscores the finding of relatively small sizes of the signal from the hyperpolarized intracellular ^133^Cs^+^. Note for convenience, we refer to this as the ‘labelled’ ^133^Cs^+^, which is a term used in some of our previous dDNP reports^25–27^. In other words, a key concept when using hyperpolarized solutes to give greatly enhanced NMR signals, is that the magnetization of the hyperpolarized spin population(s) (the ‘label’) decays to the Boltzmann thermal-equilibrium magnetization that is relatively tiny; this is tantamount to the solute becoming ‘unlabelled’ over the course of the experiment.

Figure 4B shows the labelled intracellular ^133^Cs^+^ signal scaled up 210-fold, again underscoring the smallness of the value and how this would be impossible to record had the ^133^Cs^+^ not been hyperpolarized. Since the labelled ^133^Cs^+^ becomes unlabelled by longitudinal relaxation of magnetization, application of the principle of conservation of mass enables calculation of the unlabelled concentration, and hence (from the sum of both forms) the total flux from outside to inside the RBCs. Figure 4C shows the time evolution of the labelled, unlabelled and total ^133^Cs^+^, and hence (by a scaling calculation based on our knowledge of the initial experimental conditions) the concentration of extracellular ^133^Cs^+^. Figure 4D shows that the total influx of ^133^Cs^+^ after the initial 4 s is much greater than that of the labelled form. In other words, the NMR time-course of hyperpolarized-^133^Cs^+^ signal, is only a tiny “reflection” of the actual total influx (labelled plus unlabelled) of ^133^Cs^+^.

**Figure 5:**
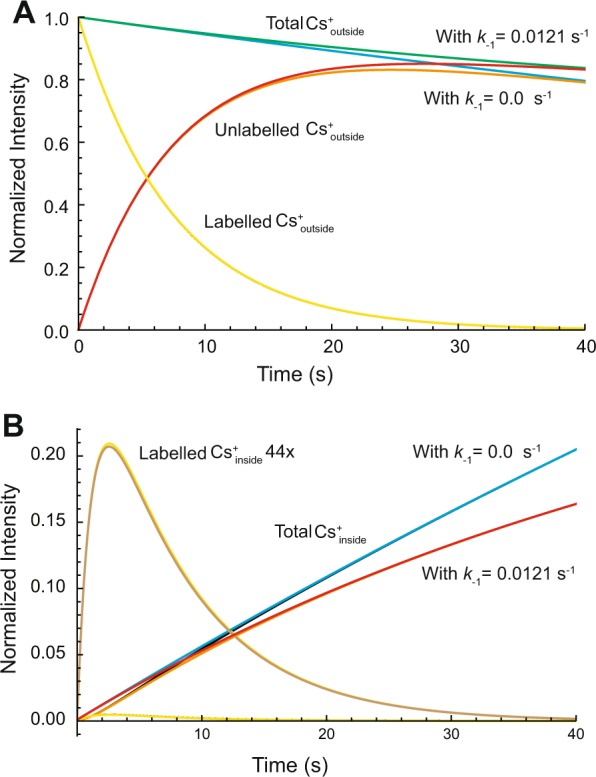
Effect of reversibility of the transport reaction on simulated time courses of ^133^Cs^+^ influx to RBCs, using the numerical integration algorithm and parameter values as for Fig. 4, but with *k*_−1_ = 0.0121 s^−1^. (**A**) Time courses of ^133^Cs^+^ outside the RBCs: total ^133^Cs^+^_outside_ when *k*_−1_ = 0.0121 s^−1^, green; total ^133^Cs^+^_outside_ when *k*_−1_ = 0.0 s^−1^, blue; unlabelled ^133^Cs^+^_outside_ when *k*_−1_ = 0.0121 s^−1^, red; unlabelled ^133^Cs^+^_outside_ when *k*_−1_ = 0.0 s^−1^, orange; labelled ^133^Cs^+^_outside_ when *k*_−1_ = 0.0 or 0.01121 s^−1^, superimposed yellow. Note, (1) the interconversion of labelled to unlabelled (simulated) cation, and (2) the slower net decline of ^133^Cs^+^ outside when the reverse reaction was allowed. (**B**), Time courses of ^133^Cs^+^ inside the RBCs: total ^133^Cs^+^_inside_ when *k*_−1_ = 0.0121 s^−1^, red; total ^133^Cs^+^_inside_ when *k*_−1_ = 0.0 s^−1^, blue; labelled ^133^Cs^+^_inside_ when *k*_−1_ = 0.0 or 0.0121 s^−1^, superimposed yellow; 44 × labelled ^133^Cs^+^_inside_ when *k*_−1_ = 0.0 or 0.0121 s^−1^, yellow and fawn, respectively. Note, (1) the slower net build-up of ^133^Cs^+^_inside_ when the reverse reaction was allowed, and (2) the almost imperceptible difference between the build-up-and-decline curves (which is nevertheless seen in the neighbourhood of the maximum) when *k*_−1_ = 0.0121 s^−1^ or 0.0 s^−1^. The value chosen for *k*_−1_ was based on the thermal time-course reported in Table S1 Row 2 that pertains to the data in Fig. 3A; in this case *k*_1_ = 0.0043 s^−1^ and *k*_−1_ = 0.0091 s^−1^, therefore to retain the same ratio of values when *k*_1_ = 0.0057, the value of *k*_−1_ became (0.0057/0.0043) × 0.0091 = 0.0121 s^−1^.

Figure 5 is shown to justify the use of the simplified model with no reverse flux for analysis of the dDNP time courses that were detected for ~40 s. Figure 5A compares the simulated time courses with and without the reverse flux. Differences in the two time courses would be unlikely to be resolved with real experimental data; notice how the ‘labelled’ time courses for the decay of ^133^Cs^+^_outside_, for which *k*_−1_ = 0.0 s^−1^ and *k*_−1_ = 0.0121 s^−1^, are completely superimposed, while the ‘unlabelled’ time courses show separation (red with *k*_−1_ = 0.0 and orange with *k*_−1_ = 0.0121 s^−1^) only after ~20 s. In addition, the time course of build-up and decline shown in Fig. 5B shows an almost imperceptible difference between the cases when *k*_−1_ = 0.0 s^−1^ and *k*_−1_ = 0.0121 s^−1^ (yellow with *k*_−1_ = 0.0 and fawn with *k*_−1_ = 0.0121 s^−1^). This justifies the use of the simpler model when fitting to dDNP data. On the other hand, the complete model with *k*_−1_ being non-zero was required for fitting the thermal time courses (Fig. 3 and Table S1) that extended over 200–1800 s.

### Cell-biological insights from the study

From the rate-data in Table S2 it is possible to estimate the number of individual ^133^Cs^+^ ions that traverse each Piezo1 trimer of an RBC per second. This is done as follows: the mean net flux of ^133^Cs^+^ was ~30 μmol (L RBC)^−1^ s^−1^ (Table S2). The volume of a normal isovolumic human RBC is 86 fL^16^; thus there are 1/(86 × 10^−15^) = 1.16 × 10^13^ RBCs L^−1^. A flux of 30 μmol (L RBC)^−1^ s^−1^ corresponds to 30 × 10^−6^ × 6.022 × 10^23^ (Avogadro’s number) ions per L of RBCs per second; or 1.6 million ions per RBC per second. This takes place through ~55 Piezo1 trimer molecules per RBC^28^, so based on our data, the *in situ* flux of Cs^+^ ions per Piezo1 molecule is ~30,000 s^−1^. The number of trimers is potentially an underestimate as the cited count per RBC ghost was higher than from the whole RBCs. Therefore, conservatively the number could by 10 times higher making the flux per trimer nearer to 3,000 s^−1^. This turnover number (*k*_cat_) is comparable to those of many enzymes; *e.g*., for enzymes in a typical metabolic pathway like the urea cycle, ornithine carbamoyl transferase (*k*_cat_ = 1,400 s^−1^) and arginase (*k*_cat_ = 4,500 s^−1^)^29,30^. Thus, fully-activated (by yoda1) Piezo1 has flux kinetics like a rapid metabolic enzyme, which is not of the same order of magnitude as the massive fluxes of many ion channels or water transporters^31^.

The second aspect of the data is the retention (or otherwise) of the membrane potential in RBCs treated with yoda1. As seen from Table S1, reporting the values of the influx and efflux rate constants, the membrane potential calculated from the values varied from ~+9 mV to ~−9 mV. There was no obvious difference in sample preparation that could account for this large range of values. However, it is possible that the concentration of 2,3-bisphosphoglycerate (2,3BPG) varied between the samples (this was not measured). 2,3BPG has a major effect on the Donnan potential, which is the basis of the membrane potential of the unperturbed RBC^18^. Therefore, this is a matter that warrants further study. Nevertheless, the fact that treatment of the RBCs with yoda1 left the RBCs in most preparations with a non-zero membrane potential implies that they remained intact. Thus, the ^133^Cs^+^ exchange was not simply via (reversible) structurally compromised membranes like with electroporated yeast cells^7^.

### Future directions

Because the *T*_1_ values of ^133^Cs^+^ both outside and inside RBCs are relatively short (<10 s) applications of ^133^Cs^+^-dDNP could be a challenge for *in vivo* studies. Such studies would have to take place in the presence of competing cations like Na^+^ and K^+^ (in blood plasma and tissues). On the other hand, the methodology constitutes (at least) a valuable tool for *in vitro* studies of membrane transport systems, such as Piezo1, as used here. Cell types other than RBCs may well show faster exchange as these cells could contain additional types of channels that mediate cation transport, increasing the likelihood of applications of the new method to such cells.

The fact that dDNP-amenable exchange rates were only achieved once yoda1 was added to RBCs means that other drug-effects on Piezo1 would have to be carried out in the presence of yoda1, or other activators as they emerge from effector-screening studies. This type of rate perturbation has already been shown in ‘thermal NMR’ studies of the inhibitory effect of GsMTx4 on human RBCs^12^. Therefore, the RBC system with this inhibitor would be amenable to further study by using ^133^Cs^+^-dDNP.

The fact that the kinetic constants estimated from both the dDNP studies and the thermal time courses were in the same range of values, implied that there were no significant (within the temporal resolution) early ‘transitional events’ like protein conformational changes. In other words, any transitional events in the transporter, if they had occurred would have done so in less than the ~1-10 s available to us for observation of the system.

Overall, we have shown how to apply dDNP for studying the rapid transport of ^133^Cs^+^ into human RBCs and estimated fluxes under various experimental conditions; and we anticipate further work with other cell-types and, potentially, tissues *in vivo*.

## Materials and Methods

### Research conduct

We confirm that all experiments were performed in accordance with relevant guidelines and regulations of The University of Sydney (Australia) and the Danish Technical University (Denmark).

### Chemicals and solutions

Analytical Reagent (AR) chemicals (including yoda1) were purchased from Sigma-Aldrich (St Louis, MO, USA) unless otherwise stated. Trityl radical OX063 was supplied by GE Healthcare (Brøndby, Denmark). Gadolinium contrast agent, Prohance, was obtained from Bracco Imaging (Milan, Italy)^7^. The saline used for RBC preparations was 154 mM NaCl, supplemented with 15 mM D-glucose as the energy source for the cells. An alternative, cation-free, isotonic medium was 300 mM sucrose, also supplemented with 15 mM D-glucose.

### Red blood cells

RBCs were prepared from blood (~50 mL) obtained by venipuncture from the cubital fossa of normal informed-consenting donors, under approval from the University of Sydney Human Ethics Committee (Institutional Review Board; Project No.: 2012/2882, Project Title: Magnetic resonance studies of red blood cell metabolism, biophysics, and cytology, and biochemical composition of plasma). The blood was anticoagulated with 15 IU mL^−1^ of porcine-gut heparin (Sigma-Aldrich). The blood was centrifuged in 50 mL Falcon tubes at 3000 × g for 5 min at 20 °C to sediment the RBCs, and the plasma and buffy coat (that contained the platelets and white blood cells) were removed by vacuum-pump aspiration. Two further washes in 1-2 volumes of saline-D-glucose (154 mM and 15 mM, respectively) were carried out with the same centrifugation-supernatant aspiration protocol as before, yielding a sample (typically ~20 mL) of *Ht* ~80% that was stored at 4 °C and typically used within 24 h.

### DNP ^133^Cs^+^ sample

The usual DNP stock solution was made of 2.6 mmol L^−1^ CsCl in a 50:50 (w/w) mixture of water and glycerol with the trityl radical OX063 and gadolinium contrast agent, Prohance, added to give final concentrations of 20 mM and 1.5 mM, respectively. The dissolution was carried out with 5 mL of physiological saline or isotonic sucrose, both with 15 mM D-glucose as the RBC substrate.

### DNP RBC sample

The RBCs used in the DNP experiments were stored at 4 °C until immediately before use. Their medium was saline-glucose. Volumes of 2.5–3.0 mL of this RBC suspension, that had been equilibrated with atmospheric oxygen by shaking with five changes of air in the tube, and with *Ht* ~0.8, were dispensed into 15 mL Falcon tubes which were filled (top mark ‘15 mL’) with a solution of 300 mM sucrose and 15 mM D-glucose. The RBC suspensions were mixed with the solution and then centrifuged at 3000 × g for 5 min at 20 °C to sediment the cells. The supernatant was aspirated as described above, and then a second aliquot of 300 mM sucrose –15 mM D-glucose was added. At this stage, yoda1 stock solution (14 mM or 28 mM) in DMSO was added to the top of the solution. The added volume varied from 10–80 μL making the yoda1 concentration ~50 μM. After screwing on the cap of the centrifuge tube the sample was briskly shaken to ensure an even distribution of the contents. After 10 min at 20 °C, the sample was again centrifuged at 3000 × g for 5 min at 20 °C to sediment the RBCs. These, with *Ht* = 0.8, were then delivered to the bottom of the precision-glass NMR tube, via a glass or PEEK tube that was attached to a disposable syringe; this was done to avoid contaminating the walls of the NMR tube with cells that might impede the movement of the piston (see Supplementary Information).

### DNP and NMR spectroscopy

Hyperpolarization of ^133^Cs^+^ was achieved in a 3.35 T HyperSense polarizer (Oxford Instruments, Oxford, UK) with the sample at 1.2 K and irradiated at the optimum microwave frequency using a 94 GHz source. Equilibrium of polarization occurred after ~1 h, following a bi-exponential build up and was typically around 50%, similar to the previously obtained values^7^.

^133^Cs NMR spectra were recorded at 52.5 MHz on a Varian Inova 400 MHz NMR spectrometer, with a shielded, vertical, narrow-bore 9.4 T, AS 400, magnet from Oxford Instruments (2006). A Varian 10-mm broad-band probe with thermal regulation was used. Spectra, including peak integrals, were extracted using MNova software (MestreLab Research S. L., Santiago de Compostela, Spain).

### Computing and regression analysis

Lists of peak integrals from NMR spectra were imported via MS Excel (Microsoft, USA) into *Mathematica* (Version 12.0.0; Wolfram Research, Champaign, ILL, USA)^32^ for regressing mathematical functions onto the data.

The two simultaneous differential equations used to model the two-site transmembrane exchange system were:

$${{\rm{Cs}}}_{{\rm{o}}}^{+}\underset{{k}_{-1}}{\overset{{k}_{1}}{\rightleftharpoons }}{{\rm{Cs}}}_{{\rm{i}}}^{+}$$

4 $$\frac{d{{\rm{Cs}}}_{{\rm{o}}}^{\ast }(t)}{dt}=-\,\frac{{{\rm{Cs}}}_{{\rm{o}}}^{\ast }(t)}{{T}_{1,{\rm{o}}}}+\frac{Ln(Cos\alpha )\,{{\rm{Cs}}}_{{\rm{o}}}^{\ast }(t)\,}{{t}_{{\rm{R}}}}-{k}_{1}{{\rm{Cs}}}_{{\rm{o}}}^{\ast }(t)+{k}_{-1}{{\rm{Cs}}}_{{\rm{i}}}^{\ast }(t)$$

5 $$\frac{d{{\rm{Cs}}}_{{\rm{i}}}^{\ast }(t)}{dt}=-\,\frac{{{\rm{Cs}}}_{{\rm{i}}}^{\ast }(t)}{{T}_{1,{\rm{i}}}}+\frac{Ln(Cos\alpha )\,{{\rm{Cs}}}_{{\rm{i}}}^{\ast }(t)\,}{{t}_{{\rm{R}}}}+{k}_{1}{{\rm{Cs}}}_{{\rm{o}}}^{\ast }(t)-{k}_{-1}{{\rm{Cs}}}_{{\rm{i}}}^{\ast }(t)$$

where ${{\rm{Cs}}}_{{\rm{o}}}^{\ast }(t)$ and ${{\rm{Cs}}}_{{\rm{i}}}^{\ast }(t)$ denote the amounts of hyperpolarized (hence the *) ^133^Cs^+^ outside and inside the RBCs, *T*_1,o_ and *T*_1,i_ denote the corresponding longitudinal relaxation time constants, outside and inside the RBCs, respectively; and *k*_1_ and *k*_−1_ denote the influx and efflux rate constants. For fitting the dDNP data, *k*_−1_ was set to 0.0, while for fitting the ‘thermal’ time courses the complete equations were used (although of course the * is redundant in this case). Note the use of the approximation $(\frac{Ln(Cos\alpha )}{{t}_{{\rm{R}}}})$ for the magnetization-relaxation effect of the RF sampling pulses that were applied every *t*_R_ = 1.0 s. Explicit accounting for the discontinuous sampling on the dDNP time courses was built into the Markov chain Monte Carlo (MCMC) script; but it was shown to make little difference to the simulated or data-regressed time courses.

The equations are applicable in a situation where there is no evidence of substrate concentration-dependence of *k*_1_ and *k*_−1_ that can occur with enzymes^26,27,33^ and membrane transport systems that operate with hyperpolarized substrates when there are large extents of reaction. This means that substrate concentrations change during the reaction to such an extent that they span the apparent *K*_m_ value(s) of the enzyme/carrier^11,25^.

For simulating the *total* fluxes of the ions (‘labelled’ plus ‘unlabelled’ populations) the formalism described in^25–27,33^ was used. This entailed using two additional differential equations and the requisite initial conditions:

6 $$\frac{d{{\rm{Cs}}}_{{\rm{o}}}(t)}{dt}=\frac{{{\rm{Cs}}}_{{\rm{o}}}^{\ast }(t)}{{T}_{1,{\rm{o}}}}-\frac{Ln(Cos\alpha )\,{{\rm{Cs}}}_{{\rm{o}}}^{\ast }(t)\,}{{t}_{{\rm{R}}}}-{k}_{1}{{\rm{Cs}}}_{{\rm{o}}}(t)+{k}_{-1}{{\rm{Cs}}}_{{\rm{i}}}(t)$$

7 $$\frac{d{{\rm{Cs}}}_{{\rm{i}}}(t)}{dt}=\frac{{{\rm{Cs}}}_{{\rm{i}}}^{\ast }(t)}{{T}_{1,{\rm{i}}}}-\frac{Ln(Cos\alpha )\,{{\rm{Cs}}}_{{\rm{i}}}^{\ast }(t)\,}{{t}_{{\rm{R}}}}+{k}_{1}{{\rm{Cs}}}_{{\rm{o}}}(t)-{k}_{-1}{{\rm{Cs}}}_{{\rm{i}}}(t)$$

and where in most experiments ${{\rm{Cs}}}_{{\rm{o}}}(t=0)=0$ and ${{\rm{Cs}}}_{{\rm{i}}}(t=0)=0$, while ${{\rm{Cs}}}_{{\rm{o}}}^{\ast }(t=0)$ is known for the composition of the hyperpolarization sample, the volume of the solution used in dissolution, and the fraction of this volume (typically 1.5 mL of 5 mL) injected into the RBC suspension.

## Supplementary information

Supplementary Information

## Data Availability

All data needed to evaluate the conclusions in the paper are presented in the paper and/or the Supplementary Information. Additional data relating to the paper may be requested from the authors.
