# Supplementary material for: Rapid zero-trans kinetics of Cs+ exchange in human erythrocytes quantified by dissolution hyperpolarized 133Cs+ NMR spectroscopy
Source: Sci Rep. 2019 Dec 23;9:19726. doi: 10.1038/s41598-019-56250-z (PMC6928147; doi:10.1038/s41598-019-56250-z)

SUPPLEMENTARY INFORMATION for

Rapid *zero-trans* kinetics of Cs^+ exchange in human erythrocytes quantified by dissolution hyperpolarized $^{133}Cs^+$ NMR spectroscopy

Philip W. Kuchel[1,2]*, Magnus Karlsson[2], Mathilde Hauge Lerche[2], Dmitry Shishmarev[3], and Jan Henrik Ardenkjaer-Larsen[2]

[1]*The University of Sydney, School of Life and Environmental Sciences, Sydney, New South Wales, Australia*

[2]*Center for Hyperpolarization in Magnetic Resonance, Department of Health Technology, Technical University of Denmark, Lyngby, Denmark*

[3]*The Australian National University, John Curtin School of Medical Research, Canberra, Australian Capital Territory, Australia*

Supplementary Discussion

Notwithstanding the theoretical and technical elegance of dDNP experiments, common undoings of experiments are inconsistencies (dispersion and broadening) in NMR line shapes that occur on rapid delivery of the solution of hyperpolarized solute into cell suspensions. This is especially relevant with reactions that occur on the 10-second time scale such as with the present experiments. So, it is imperative to have thermal and magnetic homogeneity that are optimized in this time domain. To this end, we implemented the following measures in order to catch the early stages of $^{133}Cs^+$ entry into human RBCs that had their Piezo1 ion channels activated with the selective agent, yoda1.

First, we ensured almost instantaneous thermal equilibration of the added DNP solution with the RBCs by using a counter-current heat exchanger in sample delivery.

SUPPLEMENTARY METHODS

Counter-current heat exchanger

This was based on a previous design (*1*). The central Perspex (polymethylmethacrylate; PMMA) tube carries four fine tubes (1.6-mm o.d. PEEK); it is across one of these that the internal solution is thermally equilibrated with the water circulated through a thermostated water-bath. In the new design, the manifolds at both the top and bottom of the apparatus have four holes and recesses that seat o-rings, which are compressed by tightening Nylon screws

down on them, and hence around the tubes (number 6 in the drawing in Fig. S1). This arrangement forms a water-tight seal against the water in the jacket.

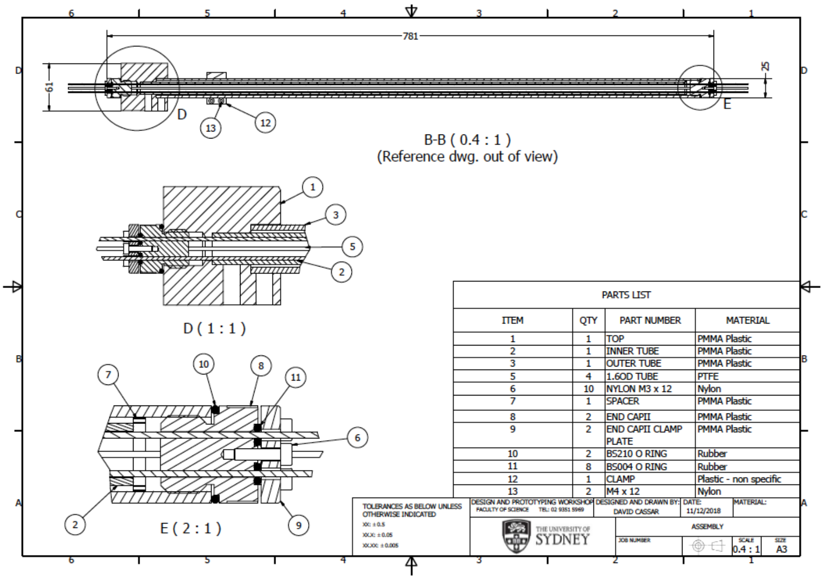

Fig. S1 | Four-tube heat exchanger, technical drawing. Top, is an overview of the 25-mm o.d. tube that went inside the bore of the NMR magnet. Centre, is a cross-section of the upper manifold showing the inlet and outlet for the water from the circulating water bath. Bottom, shows details of the o-rings (black-disc cross-sections) that are compressed onto the thin tubes making the system water tight. The drawing was made in Autodesk Inventor (Sydney, NSW, Australia) by David Cassar (see Acknowledgements).

The heat exchanger was circulated with water from a bath that was regulated at 37°C; and based on bench tests the DNP-solution entered RBC suspensions at this temperature.

Second, the uniformity of mixing the DNP-solution would be optimized by withdrawing the open end of the delivery tube (at a constant rate), up through the full extent of the suspension of RBCs. Furthermore, magnetic homogeneities in the sample would be reduced by removing the solution-delivery tube from the active volume of the RF coil in the NMR probe. Both outcomes were achieved with a piston that moves inside the precision, thin-walled glass NMR tube [Serial Number 513-7PP, outside diameter (o.d.) 10 mm, inside diameter (i.d.) 9.03 ± 0.01 mm, Wilmad, Buena, NJ, USA] that contains the cell suspension.

Piston

This is a cylinder that was turned, to specified dimensions in a lathe, from the stable polymer PEEK. It fits snugly inside the 20.32-cm (8”) long glass, 10-mm o.d. NMR tube (see Fig. S2). Three Teflon tubes are connected to it; the central one delivers the DNP solution, and the two lateral ones carry air that is used to displace the piston up and down inside the NMR tube. The fins-and-grooves serve as a hydraulic seal, between the piston and the glass tube, which is

enhanced by applying a drop of water to the piston prior to inserting it in the NMR tube. Each piston was matched to a particular precision NMR tube by polishing with 800-grit Emery paper, giving a wall clearance of ~5 μm.

Fig. S2 | PEEK piston. A cylinder, 23 mm long and 9.02 mm o.d. with 11 × 0.5-mm wide fins separated by 0.5-mm grooves 1.5 mm deep. The lower and upper smooth sections are 4 mm and 8 mm long, respectively. The solution-delivery tube of 1.6-mm o.d. Teflon tube passes along the axis of the cylinder, and two parallel holes carry the same type of Teflon tube to deliver piston-displacing air.

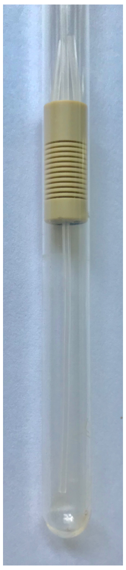

Piston movement within the NMR tube

The experiment begins by adding the suspension of RBCs to a 10-mm glass NMR tube which is then lowered into the probe in the spectrometer magnet. Typically, the volume of cells was 1.5 mL with *Ht* = 0.8. Fig. S3 is a schematic description of the sequence of events used in the subsequent experiment; these are numbered (1- 7). The piston is connected to three tubes from the heat exchanger at the top end of which are three separate syringes in a special drive system (see below). Thus, the syringes, tubes, and air-space below the piston, form a closed system, such that air pushed from a syringe, or drawn back into it, will cause the piston to move up and down in the NMR tube. (1) Shows the piston positioned so that the outlet of the delivery tube is just clear of the top of the RBC suspension. Once the solution of hyperpolarized solute is ejected from the HyperSense (generator of the hyperpolarized state) it is ready for injection into the RBC suspension. (2) Shows the displacement of air in the delivery tube by the solution of hyperpolarized solute (coloured dark blue) pushed in by its syringe; and concomitantly the piston is displaced upwards by the air that has been displaced from the delivery tube. The volume of air in the dead-space is pre-measured and the volume of solution that is injected just filled this space, (3). Then, the delivery tube, which is now full of solution, is driven downwards, by withdrawing sufficient air from under the piston (4) with its syringe (via one of the two tubes that opens on the under face of the piston) to make the delivery-tube outlet rest on the bottom of the NMR tube, (5). Now the NMR spectrometer is triggered to begin

acquiring spectra, as the solution of hyperpolarized solute and an equal volume of air are injected from their respective syringes; the solution (dark blue) enters from the central delivery tube, while air enters just below the piston. Both injected volumes displace the piston upwards such that, at the end of delivery, the outlet of the central tube sits on the top of the mixed sample. It is as if the solution has been distributed in layers in the RBC suspension making the spread of solution around the RBCs, very uniform.

Syringe-drive sequence

The various stages in the movement of the piston and solution-delivery tube are represented in Fig. S4. The controlled delivery is achieved with three syringes operated in sequence as shown in the figure and explained in the caption.

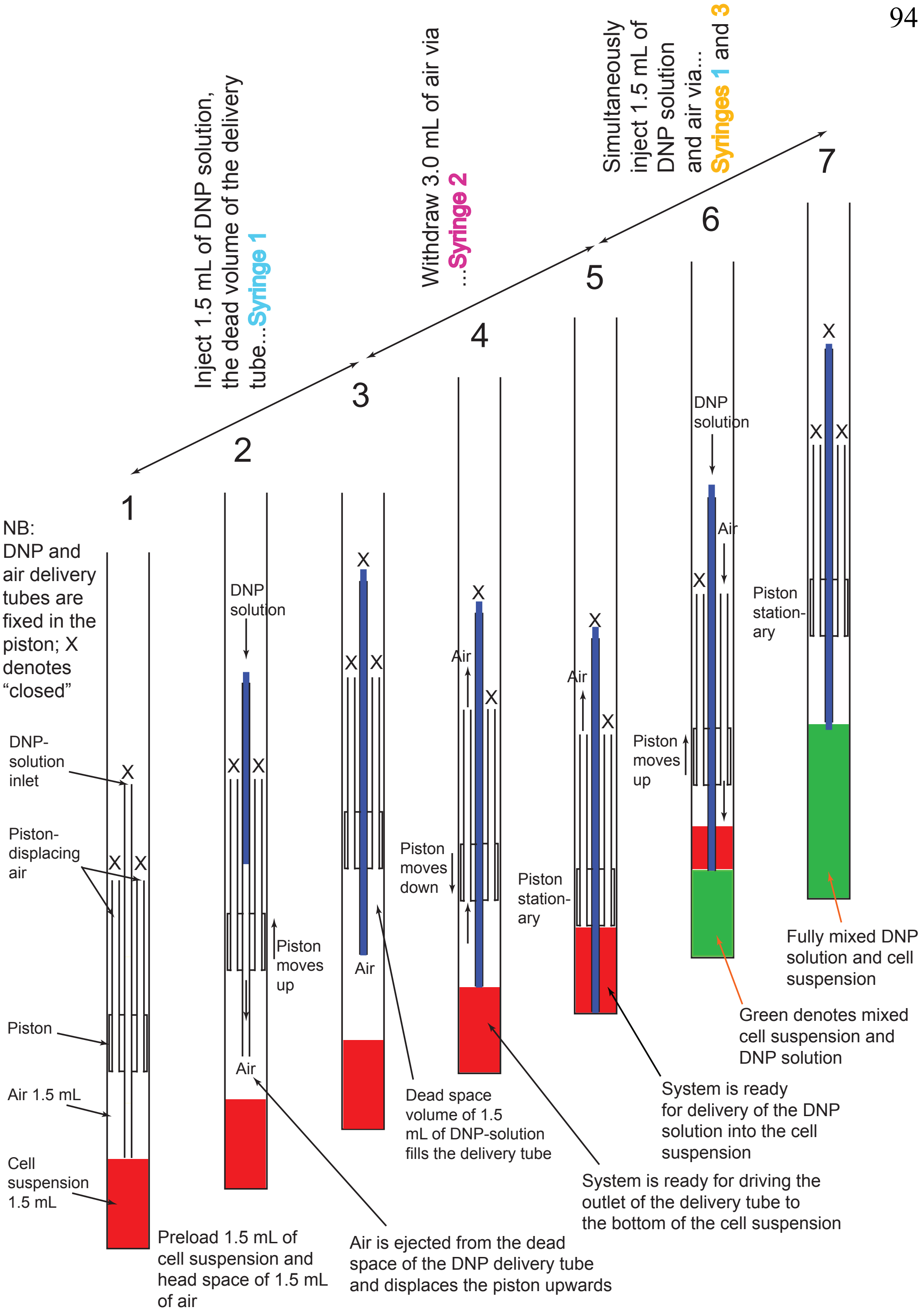

Fig. S3 | Sequence of events used in the delivery of a solution of hyperpolarized solute into an RBC suspension. The delivery occurs over ~6 s and leads to rapid and uniform mixing with the RBC suspension, while the delivery tube is withdrawn to the top of the fully mixed sample. The three “syringe” labels refer to the syringes in the delivery rack shown in Figs S4 – S6. Key: red signifies a suspension of RBCs, dark blue signifies the DNP-solution, and green the mixture of this solution and the RBCs.

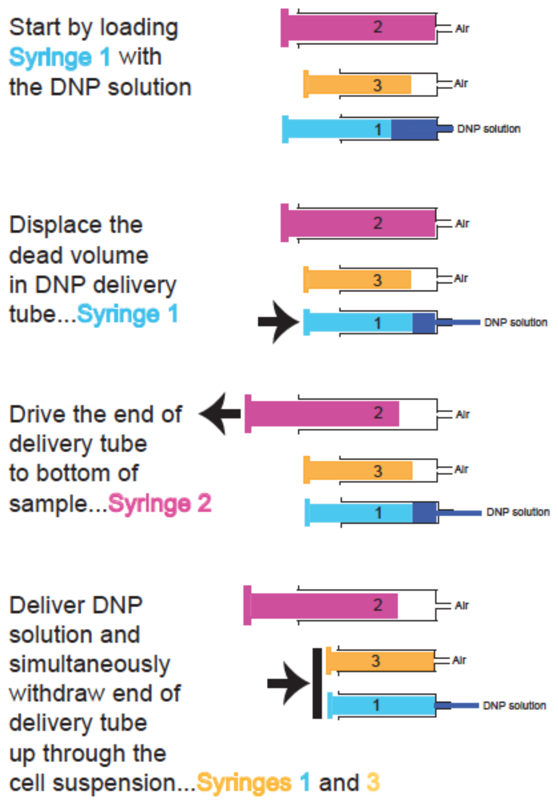

Fig. S4 | Main stages in the sequential operation of the three syringes of the DNP-solution delivery system. The initial state of the syringe system is with syringe #1 (light blue plunger) loaded with the solution of hyperpolarized solute (dark blue) via a three way tap from a receiving receptacle (a 50 mL syringe barrel); and syringe #2 (pink plunger) has its plunger fully depressed, while syringe #3 (orange plunger) has a volume of air that is equal to the volume of solution of hyperpolarized solute that will be injected into the RBC suspension. The next stage (second panel) is to inject this solution down through the heat exchanger (right facing arrow), displacing air, and consequently displacing the piston upwards, in the NMR tube [Fig. S3 (1-3)]. The third panel from the top shows the pink plunger of syringe #2 being withdrawn, sucking air from under the piston and driving the end of the solution-delivery tube to the bottom of the NMR tube [Fig. S3 (4 and 5)]. The bottom panel indicates the simultaneous injection (black right-facing arrow) of the solution of hyperpolarized solute (dark blue) from syringe #1 (light blue piston) and air from syringe #3. This uniformly adds the solution while uniformly moving the outlet of the tube up through the suspension of cells.

Double-displacement delivery device (D4 apparatus)

Figures S5 is a labelled rendering of the technical drawing used when making the three-syringe injection system. Figure S6 shows the device in its operational form.

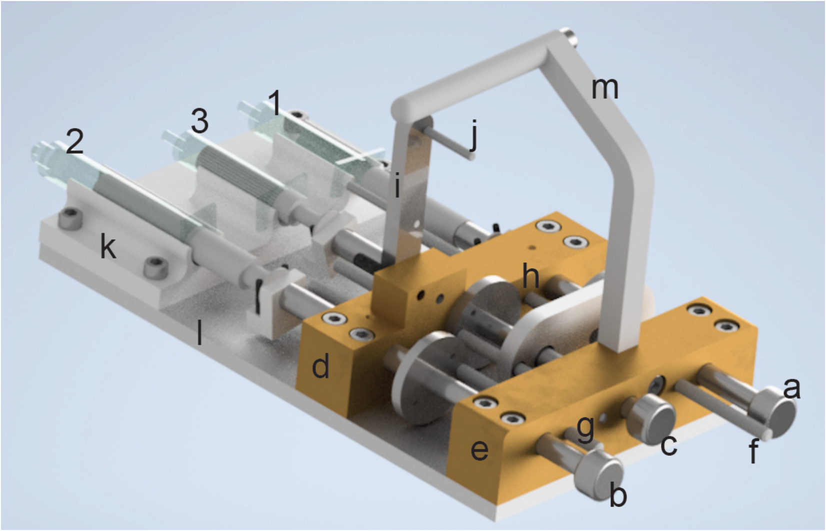

Fig. S5 | Rendered technical drawing of the three-syringe apparatus. It achieves rapid, bubble-free uniform mixing of DNP solutions in RBC suspensions. Syringe #1 of Fig. S4 is on the right, syringe #2 is on the left, and syringe #3 is in the middle. The push-pieces (stainless steel, a, b, c) pass through two brass blocks (d and e); and their displacement, and hence that of the attached pistons is accurately adjusted with screws (f, g, and h) in threaded holes in the brass blocks together with locking nuts (not shown). The stop-screws act against circular discs that are positioned between the two brass blocks (d and e). The vertically position bar (i) carries a stop screw (j) that limits the travel of syringe #1 when it is rotated to its rest position along the middle brass bar (d). It is moved up when the simultaneous injections from syringes #1 and #3 is made to take place in the final delivery stage of the sequence. The syringe holder (k) was 3D-printed from ABS plastic. The syringes are disposable, of polythene with Neoprene seals on their plungers, Luer locks on their outlets, and volumes of 5, 10 and 5 mL, respectively. The base (l) is 10 mm aluminium and the horizontal carrying handle (m) is stainless steel with an aluminium upright. The drawing was made in Autodesk Inventor (Sydney, NSW, Australia) by David Cassar (see Acknowledgements).

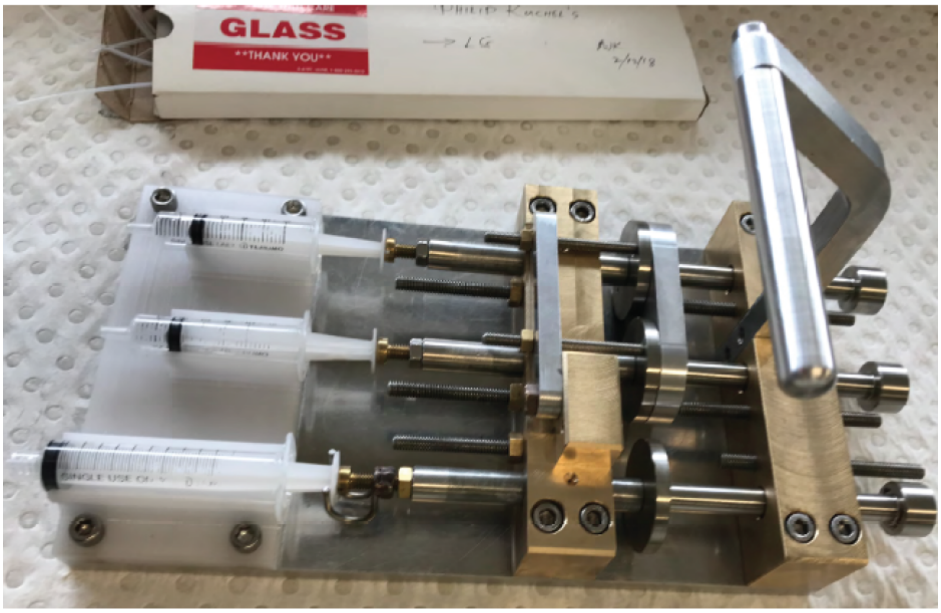

Fig. S6 | The three-syringe apparatus. The various components are described in Fig. S5, and note the locking hex-nuts on the stop-screws, which are not shown in Fig S5.

Notes on technical aspects of conducting the experiments

Transfer of hyperpolarized solution to the D4 system: The solution was delivered from the HyperSense via a ~3-m (*i.d.*, 1 mm Teflon) transfer tube, to a 20 mL syringe barrel that was connected to a 3-way tap on the Luer-lock fitting of Syringe 1 (Figs S4 and S5). From there the solution was drawn into Syringe 1 until the outside end of the plunger was stopped by the pre-set 'stop' on the push-rod, **a** in Fig. S6. The 3-way tap was rotated to enable injection of the contents of Syringe 1 down into the sample, in the sequence of syringe actions that are described in Figs S3 and S4.

D4 system placement: The D4 assembly (Fig. S6) rested on top of the Ultrashield 9.4 T superconducting magnet, constrained between one of the cryogen-filling towers and the current-energizing tower, held in place by a retort clamp.

Actuation: The push-rods and hence the syringes were/are operated manually. There is clearly scope for the D4 system to be automated with pneumatic, hydraulic, or stepper motor actuation.

Timing of each event: The total time for hyperpolarized-sample delivery (from the time when the dissolution valve was heard to open), was regularly ~10 s. The critical time, which determines the quality of the spectra acquired in the initial stage of the reaction, is Stages 5-7 in Fig. S3, whereby the hyperpolarized solution mixes with the suspension of cells. This typically took place over ~2 s.

Uniformity of mixing: This was assessed by visual inspection of the sample using a solution of blue dye (Coomassie blue) in water delivered into clear water, in multiple dummy runs done outside the magnet. In actual NMR experiments, the quality of sample mixing was assessed by the reproducible symmetry, and width, of the resonances in the recorded spectra over several seconds to minutes. This was followed by inspection of the sample at the end of the time course, which typically confirmed a uniform mixture.

SUPPLEMENTARY RESULTS

'Thermal' time course analysis. (Example *Mathematica* script that generated data for Fig. 3A, row 2, is given below.)

Table S1. Non-dDNP transport time courses of $^{133}Cs^{+}$ entry into RBCs at 37°C.

Experiment number[a]	RBC treatment with yoda1[b]	Details delivery	Initial $[^{133}Cs^+]$ (mM)	k_1 ($10^3 \times s^{-1}$)	k_{-1} ($10^3 \times s^{-1}$)	Membrane potential (mV)
1	80 µL of 14 mM	D4[c]	16.7	3.2 ± 0.3	7.8 ± 0.7	+4.0
2 Fig. 3A	80 µL of 14 mM	D4	33.3	4.3 ± 0.1	9.1 ± 0.2	-0.1
3	40 µL of 14 mM	D4	33.3	3.7 ± 0.3	5.3 ± 1.0	-9.0
4	40 µL of 14 mM	D4	33.3	4.3 ± 0.1	8.7 ± 0.4	-0.9
5	40 µL of 14 mM	D4	33.3	3.5 ± 0.1	9.5 ± 0.2	+7.5
6 Fig. 3B	40 µL of 14 mM	Injected RBCs into DNP solution	33.3	4.3 ± 0.2	13.4 ± 0.7	+9.9
Weighted means				4.0 ± 0.5	9.1 ± 1.3	+1.8[d]

[a]These numbers are keyed to the laboratory records as follows:1, (June 3 #2); 2, (June 18 #1; Fig 3A); 3, (June 18 #2); 4, (June 19 #1); 5, (June 19 #2); 6, (June 20; Fig 3B).

[b]80 µL of 14 mM yoda1 in DMSO was added to 3.0 mL RBCs (Ht = 0.8). See Materials and Methods for further interpretation of this dose per RBC.

[c]The D4 system is the three-piston delivery system described above.

[d]Calculated using the equilibrium expression: $[Cs^+]_{out}/[Cs^+]_{in} = k_{-1}$ Inside volume/(k_1 Outside volume) = (k_{-1}/k_1)(32/68), the latter being the relative volume fractions of water in the Ht = 0.4 samples.

Table S2. First order kinetic rate constants of hyperpolarized $^{133}Cs^+$ uptake into RBCs from various initial concentrations, at 37°C. The yoda1 added to the RBCs, to activate their Piezo1 mechanosensitive cation channels, was also varied systematically. The longitudinal relaxation times for the $^{133}Cs^+$ inside and outside the RBCs were estimated; the values were essential to be known in making the estimates of the rate constant, and *vice versa*. This was done simultaneously in the MCMC fitting procedure.

Experiment[a]	Yoda1 (µL)[b]	$[^{133}Cs^+]$ mM[c] (Dilution by 1.5/1.8 = 0.8333)	$T_{1,o}$ (s)	$T_{1,i}$ (s)	k_1 ($10^3 \times s^{-1}$)	$^{133}Cs^+$ influx [µ mol (L RBC)$^{-1}$ s^{-1}]
1	20 (equiv; 19 µM)	51 (42.5)	8.4 ±	2.3 ±	0. 4 ± 0.04	42 ± 4

2	20 (equiv; 19 μM)	40 (33.3)	10.3 ±	1.8 ±	0.5 ± 0.05	42 ± 4
3	20 (equiv; 19 μM)	10 (8.3)	10.5 ±	2.1 ±	0.9 ± 0.1	19 ± 2
4	20 (equiv; but different RBC preparation protocol)	20 (16.7)	17 ± 2	1.2 ± 0.4	0.8 ± 0.2	70 ± 20
5	20 (equiv; 19 μM)	20 (16.7)	8.3 ± 0.1	1.6 ± 0.3	0.8 ± 0.1	33 ± 3
6	40 (38 μM)	4 (3.3)	9.2 ± 0.1	1.4 ± 0.1	5.8 ± 0.5	48 ± 5
7	40 (38 μM)	3.9 (3.2)	10.6 ± 0.1	1.6 ± 0.2	3.5 ± 0.5	28 ± 3
8	40 (38 μM)	4 (3.3)	9.7 ± 0.1	1.4 ± 0.1	2.6 ± 0.2	22 ± 2
9	80 (77 μM)	4 (3.3)	9.6 ± 0.5	1.9 ± 0.2	3.6 ± 0.3	30 ± 3
10 Fig. 2A	80 (77 μM)	4 (3.3)	8.9 ± 0.1	1.2 ± 0.1	5.7 ± 0.6	47 ± 5
11	80 (77 μM)	4 (3.3)	9.6 ± 0.3	1.8 ± 0.1	3.3 ± 0.2	27 ± 3
12	80 (77 μM)	1.9 (1.6)	9.4 ± 0.3	2.0 ± 0.1	3.1 ± 0.2	12 ± 1
Unweighted mean, Rows 6-12			9.6 ± 0.2	1.6 ± 0.1		31 ± 3
13 Fig. 2C	40 (38 μM)	40 (33.3)	13.6 ± 1.5	2.4 ± 0.6	0.8 ± 0.2	67 ± 7
14	40 (38 μM)	3 (2.5)	9.2 ± 0.3	0.72 ± 0.3	0.6 ± 0.2	3.7 ± 0.4
15 Fig. 2B	40 (38 μM)	3 (2.5) on 33 mM non-hyperpolarized CsCl	9.6 ± 0.2	3.0 ± 0.7	2.4 ± 0.6	15 ± 2
16	40 (38 μM)	3 (2.5)	9.4 ± 0.4	2.6 ± 0.3	2.5 ± 0.3	16 ± 2
17	40 (38 μM)	2 (1.7)	7.5 ± 0.1	0.9 ± 0.2	1.1 ± 0.4	4.6 ± 0.5
18	40 (38 μM)	2 (1.7)	13.2 ± 2.0	0.9 ± 0.2	3.4 ± 0.9	14 ± 1

[a]These numbers are keyed to the laboratory records as follows: 1, DNP 190430_4 NLLS; 2, DNP 190430_3 NLLS; 3, DNP 190430_2 NLLS; 4, DNP 190430-1x; 5, DNP 190429_2 MCMC E; 6, DNP 190529-2x A; 7, DNP#1 190530-2x A; 8, DNP#2 190530-2x; 9, DNP#3 190530-4x; 10, DNP#4 190530-4x Fig. 2A; 11, DNP#5 190530-4x; 12, DNP#6 190530-4x; 13, DNP of 25 June 1st MCMC Fig. 2C 2nd; 14, DNP of 25 June 4th MCMC;

15, DNP of 26 June 1st MCMC Fig. 2B; 16, DNP of 26 June 2nd MCMC; 17, 14. DNP of 26 June 3rd MCMC; 18, DNP of 26 June 4th MCMC.

[b]80 µL of 14 mM yoda1 in DMSO was added to 3.0 mL RBCs (*Ht* = 0.8). See Materials and Methods for further interpretation of this dose per RBC.

[c]The first number is the concentration of the DNP solution delivered from the HyperSense. This is diluted by the interstitial medium in the RBC suspension, of *Ht* = 0.8, to define the initial concentration of cation to which the cells are exposed.

'Data regression analysis for the 'thermal' time courses: *Mathematica* script using ParametricNDSolveValue and NonlinearModelFit.

This specific example generated the data for Fig. 3A, and the parameter estimates are given in row 2 of Table S1.

…attached below.

Data regression analysis for the dDNP time courses: *Mathematica* script using an MCMC algorithm.

This specific example generated the data for Fig. 2C, and the parameter estimates are given in row 13 of Table S2. However, note that re-running such a random-number generator-based regression procedure yields output values that show slight differences from one run to the next as in this case.

…attached below.

Thermal timecourse...ran for 1800 s but cut the data analysis off at 600 s...PWK 18/6/19
Remove the effect of RBC settling by fitting a straight line to the signal decay (outside) and signal drift up (inside) for the section from 400 to 600 s.

In[162]:=
```
dataDir = SetDirectory["/Users/philipwilliamkuchel/Desktop/Thermal CsCl_RBC/"];

data1 = Import["20190618_CsCl_33mM_RBC_thermal 1.xlsx"][[1]];
```

In[164]:=
```
timeData1 = Drop[
   Transpose[Drop[Import["20190618_CsCl_33mM_RBC_thermal 1.xlsx"][[1]], 1]][[
     1]] + 10, -120];
outsideData1 = Drop[Transpose[Drop[
      Import["20190618_CsCl_33mM_RBC_thermal 1.xlsx"][[1]], 1]][[2]], -120];
insideData1 = Drop[Transpose[Drop[
      Import["20190618_CsCl_33mM_RBC_thermal 1.xlsx"][[1]], 1]][[3]], -120];
totalCM = outsideData1 + insideData1;
normOutsideData1 = outsideData1 / totalCM;
normInsideData1 = insideData1 / totalCM;
outside = Transpose[Join[{timeData1}, {normOutsideData1}]];
inside = Transpose[Join[{timeData1}, {normInsideData1}]];
Length[totalCM]
```

Out[172]= 60

In[173]:=
```
(* Here we fit a straight line to the 400 -
  600 s data with a view to subtracting the line from the data from t = 0 *)

truncatedoutside =
  Transpose[Join[{Drop[timeData1, 39]}, {Drop[normOutsideData1, 39]}]];
truncatedinside = Transpose[
   Join[{Drop[timeData1, 39]}, {Drop[normInsideData1, 39]}]];
outLine = Fit[truncatedoutside, {1, x}, x];
inLine = Fit[truncatedinside, {1, x}, x];

(* Use a Pure Function for the "correction" operation *)

corroutside = {#[[1]], #[[2]] + (0.0001034676427415176` #[[1]])} & /@ outside;
corrinside = {#[[1]], #[[2]] - (0.0001034676427415176` #[[1]])} & /@ inside;
```

In[179]:=
```
gph1 = ListPlot[corroutside, PlotRange → {{0, 600}, {0, 1}}, Joined → True]
gph2 = ListPlot[corrinside, PlotRange → {{0, 600}, {0, 1}}, Joined → True]
```

Out[179]=

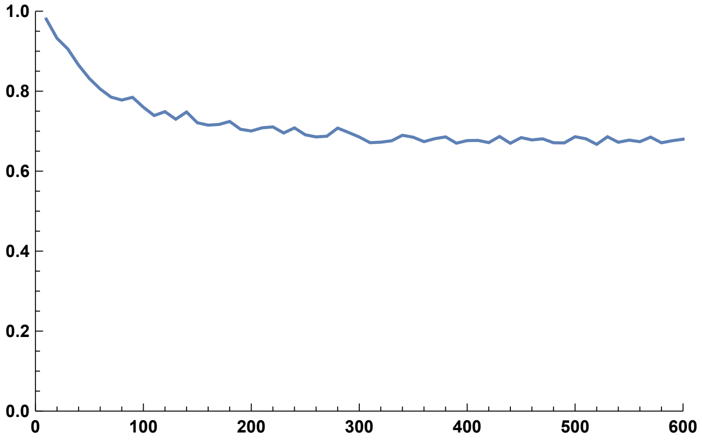

Out[180]=

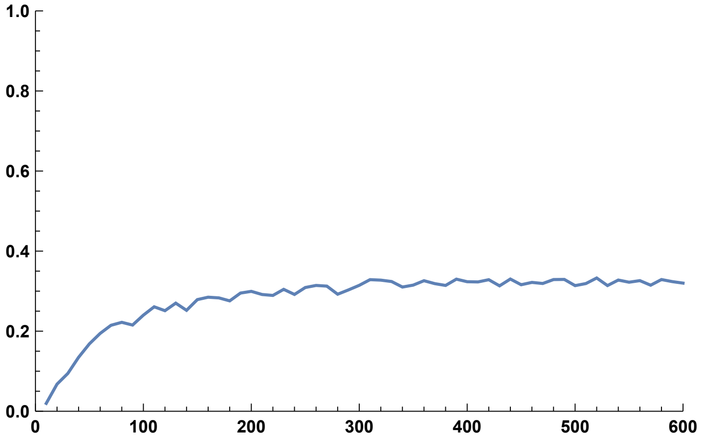

In[181]:=
```
(* For the rest of the analysis, to save having to rename the data lists,
name the corrected outside and inside data by their original name *)

outside = corroutside;
inside = corrinside;
```

In[183]:=
```
marker1 = Graphics[{Blue, Disk[]}];
marker2 = Graphics[{Red, Disk[]}];
gph2 = ListPlot[outside, Joined → False,
  PlotStyle → {Black, 0.1}, AxesStyle → Directive[Black, Thick, 14],
  PlotMarkers → {marker1, 0.04}, PlotRange → {{0, 600}, {0, 1}}]
gph3 = ListPlot[inside, Joined → False, PlotStyle → {Black, 0.1},
  AxesStyle → Directive[Black, Thick, 14],
  PlotMarkers → {marker2, 0.04}, PlotRange → {{0, 600}, {0, 1}}]
```

Out[185]=

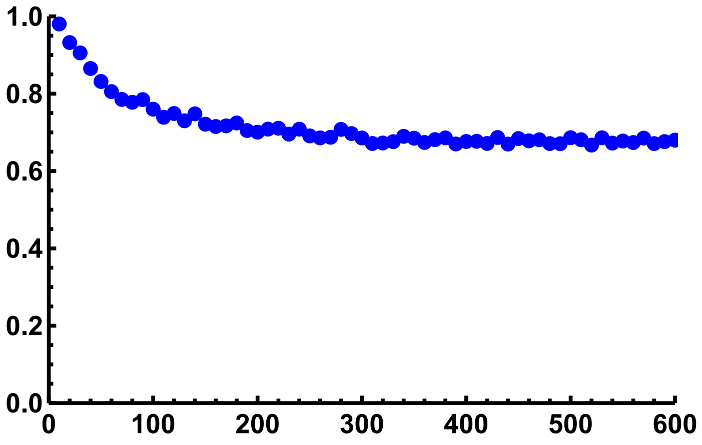

Out[186]=

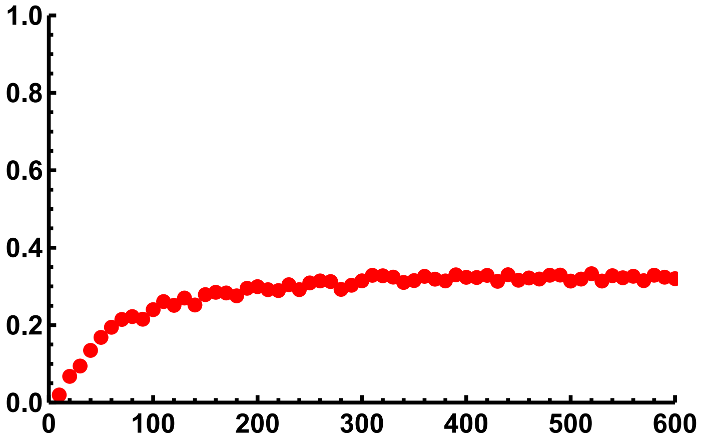

Obtain numerical solutions of the system of differential equations

In[187]:=
```
ClearAll[sol, co, ci, co0, ci0, km1, k1];

(* The co and ci denote amounts of hyperpolarized 133
 Cs+ outside and inside the RBCs, respectively *)

sol := NDSolve[{
   co'[t] == km1 ci[t] - k1 co[t] , co[0] == co0,
   ci'[t] == -km1 ci[t] + k1 co[t], ci[0] == ci0},
  {co, ci},
  {t, startTime, endTime}]
```

In[189]:=
```
(* Some starting guesses *)
co0 = 1.0;
ci0 = 0.0;
k1 = 0.0043;
km1 = 0.0091;

startTime = 0.0;
endTime = 600.0;

gph4 = Plot[Evaluate[co[t] /. sol],
   {t, startTime, endTime}, PlotStyle → {Black, 0.1}, PlotRange → All];
gph5 = Plot[Evaluate[ci[t] /. sol], {t, startTime, endTime},
   PlotStyle → {Black, 0.1}, PlotRange → All];

Show[{gph2, gph4, gph3, gph5}, PlotRange → {{0, 607}, {0, 1}}]
```

Out[197]=

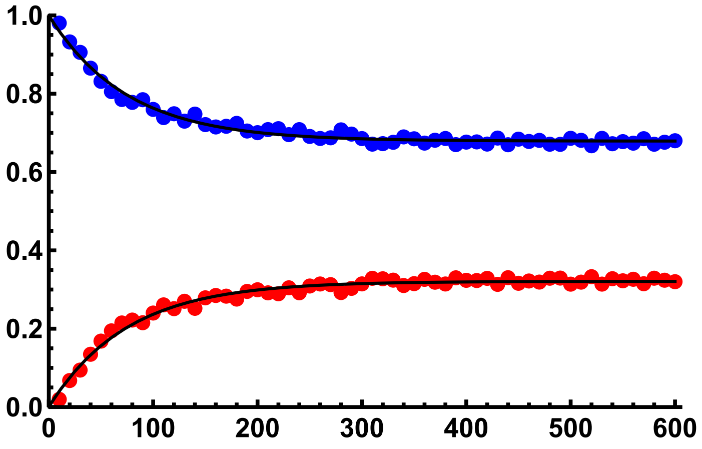

Now numerically solve the DEs so that they are parametrized with respect to the fitting parameters, using the function ParametricNDSolveValue

In[198]:=
```
ClearAll[sol1, co, ci, co0, ci0, km1, k1, startTime, endTime];

startTime = 0.0;
endTime = 600.0;

sol2 = ParametricNDSolveValue[{
    co'[t] == -k1 co[t] + km1 ci[t] , co[0] == co0,
    ci'[t] == -km1 ci[t] + k1 co[t], ci[0] == ci0}, ci,
   {t, startTime, endTime}, {k1, km1}]
(* Here I have selected the solution for 133Cs+ inside the RBCs, viz., ci *)

sol1 = ParametricNDSolveValue[{
    co'[t] == km1 ci[t] - k1 co[t] , co[0] == co0,
    ci'[t] == -km1 ci[t] + k1 co[t], ci[0] == ci0}, co,
   {t, startTime, endTime}, {k1, km1}]
(* Here I have selected the solution for 133Cs+ outside the RBCs, viz., co *)
```

Out[201]= ParametricFunction[Expression: ci Parameters: {k1, km1}]

Out[202]= ParametricFunction[Expression: co Parameters: {k1, km1}]

Evaluating the numerical solution with numerical values of co(0), ci(0), k1, and km1 that yield an approximate function for ci

In[203]:=
```
co0 = 1.0;
ci0 = 0.0;
(* Guesstimate the values of the two parameters *)
k1Guess = 0.0043;
km1Guess = 0.0091;

(* sol1 is evaluated with the chosen set of two parameter values *)
y1 = sol1[k1Guess, km1Guess]
y2 = sol2[k1Guess, km1Guess]
```

Out[207]= InterpolatingFunction[Domain: {{0., 600.}} Output: scalar]

Out[208]= InterpolatingFunction[Domain: {{0., 600.}} Output: scalar]

In[209]:=
```
gph6 = Plot[y2[t], {t, startTime, endTime}, PlotRange → All];
gph7 = ListPlot[{inside}];
Show[{gph6, gph7}]
gph61 = Plot[y1[t], {t, startTime, endTime}, PlotRange → All];
gph71 = ListPlot[{outside}];
Show[{gph61, gph71}]
```

Out[211]=

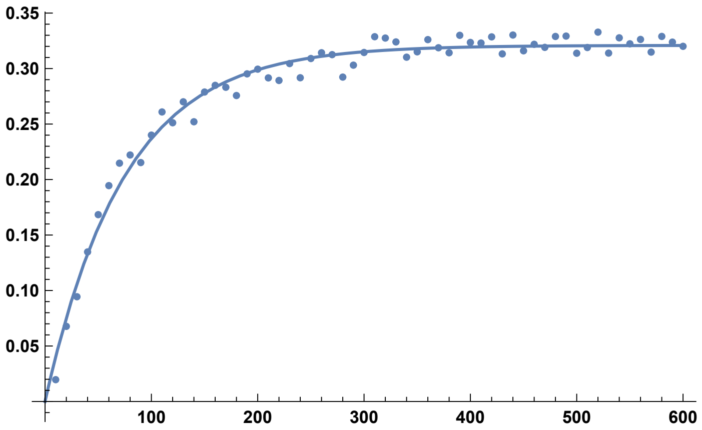

Out[214]=

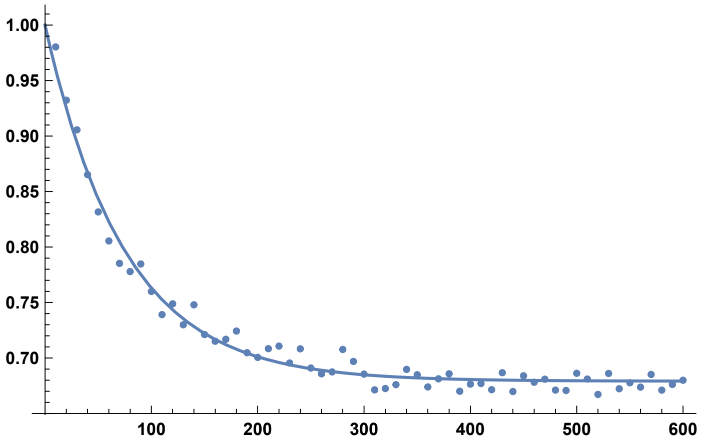

In[215]:=
```
bestFit = FindFit[inside, sol2[k1, km1][t],
  {{k1, k1Guess}, {km1, km1Guess}}, t, Method → Automatic]

bestFit1 = FindFit[outside, sol1[k1, km1][t],
  {{k1, k1Guess}, {km1, km1Guess}}, t, Method → Automatic]
```

Out[215]= {k1 → 0.00429653, km1 → 0.00907892}

Out[216]= {k1 → 0.00429653, km1 → 0.00907892}

```
In[217]:= t1 = Table[1, {Range[Length[outside]]}]; (* Make a list of "1"s *)
          t2 = Table[2, {Range[Length[inside]]}]; (* Make a list of "2"s *)
          appDataCo = Transpose[Join[{t1}, outside // Transpose]
            ]; (* Open up (transpose) the Co vs
            time data ready to prepend a "1" into each 2-tuple *)
          appDataCi = Transpose[Join[{t2}, inside // Transpose]
            ]; (* Open up (transpose) the Ci vs
            time data ready to prepend a "2" into each 2-tuple *)
          casedData = Join[appDataCo, appDataCi];
           (* Join the two data sets *)

In[222]:= bestFit2 =
           FindFit[casedData, Switch[case, 1, sol1[k1, km1][t], 2, sol2[k1, km1][t]],
            {{k1, 0.004}, {km1, 0.006}}, {case, t}, Method → Automatic]
          {bestk12, bestkm12} = {k1, km1} /. bestFit2

Out[222]= {k1 → 0.00429653, km1 → 0.00907892}

Out[223]= {0.00429653, 0.00907892}

In[224]:= bestFit2[[1]][[2]]
          bestFit2["EstimatedVariance"]
          bestFit2["ParameterTable"]
          bestFit2["ANOVATable"]

Out[224]= 0.00429653

Out[225]= {k1 → 0.00429653, km1 → 0.00907892}[EstimatedVariance]

Out[226]= {k1 → 0.00429653, km1 → 0.00907892}[ParameterTable]

Out[227]= {k1 → 0.00429653, km1 → 0.00907892}[ANOVATable]

In[228]:= gph9 = Plot[sol1[bestk12, bestkm12][t], {t, startTime, endTime}, PlotRange → All];
          gph10 = ListPlot[{outside}];
          Show[{gph9, gph10}]
```

Out[230]=

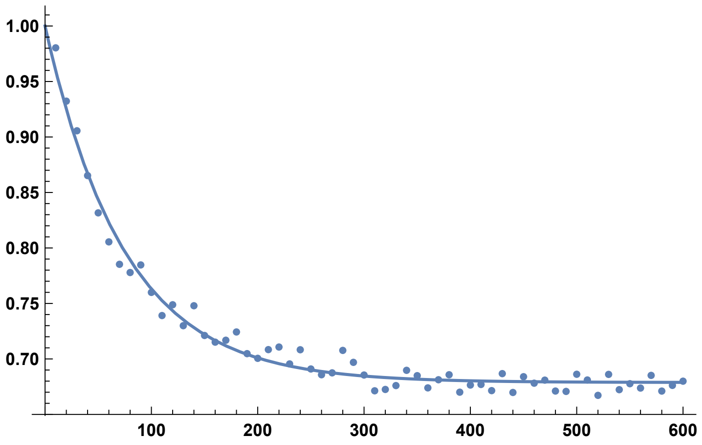

In[231]:=
```
gph11 =
  Plot[sol2[bestk12, bestkm12][t], {t, startTime, endTime}, PlotRange → All];
gph12 = ListPlot[{inside}];
Show[{gph11, gph12}]
```

Out[233]=

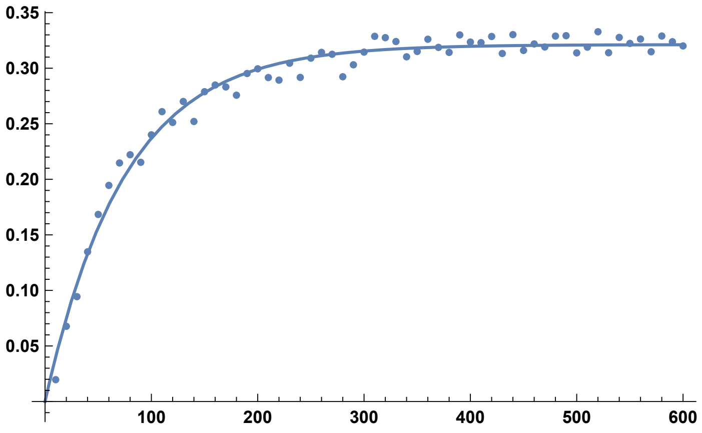

In[234]:=
```
Clear[k1, km1];
bestFit2 = NonlinearModelFit[casedData,
  If[case == 1, sol1[k1, km1][t], sol2[k1, km1][t]]
  (*Switch[case,1,sol1[k1,km1][t],2,sol2[k1,km1][t]]*),
  {{k1, 0.003}, {km1, 0.003}}, {case, t}]
{bestk12, bestkm12} = {k1, km1} /. bestFit2
```

Out[235]= FittedModel[If[case == 1, sol1[0.00429653, 0.00907892][t], sol2[0.00429653, 0.00907892][t]]]

... **ReplaceAll**: {FittedModel[If[case == 1, sol1[0.00429653, 0.00907892][t], sol2[0.00429653, 0.00907892][t]]]} is neither a list of replacement rules nor a valid dispatch table, and so cannot be used for replacing.

... **Set**: Lists {bestk12, bestkm12} and {k1, km1} /. FittedModel[If[case == 1, sol1[0.00429653, 0.00907892][t], sol2[0.00429653, 0.00907892][t]]] are not the same shape.

... **ReplaceAll**: {FittedModel[If[case == 1, sol1[0.00429653, 0.00907892][t], sol2[0.00429653, 0.00907892][t]]]} is neither a list of replacement rules nor a valid dispatch table, and so cannot be used for replacing.

Out[236]= {k1, km1} /. FittedModel[If[case == 1, sol1[0.00429653, 0.00907892][t], sol2[0.00429653, 0.00907892][t]]]

In[237]:=
```
bestFit2[[1]][[2]]
bestFit2["EstimatedVariance"]
bestFit2["ParameterTable"]
bestFit2["ANOVATable"]
```

Out[237]= {k1 → 0.00429653, km1 → 0.00907892}

Out[238]= 0.0000882994

Out[239]=

	Estimate	Standard Error	t-Statistic	P-Value
k1	0.00429653	0.0000720049	59.6699	5.47691×10^{-90}
km1	0.00907892	0.000178439	50.8796	3.99356×10^{-82}

Out[240]=

	DF	SS	MS
Model	2	36.1365	18.0683
Error	118	0.0104193	0.0000882994
Uncorrected Total	120	36.1469	
Corrected Total	119	6.14694	

Preparation for figures in the manuscript...PWK Saturday 29/6/19

In[241]:=
```
marker1F = Graphics[{Black, Disk[]}];
marker2F = Graphics[{Black, Disk[]}];

gph9F = Plot[sol1[bestk12, bestkm12][t],
   {t, startTime, endTime}, PlotStyle → {Black, 0.1},
   AxesStyle → Directive[Black, Thick, 14], PlotRange → All];

gph10F = ListPlot[{outside}, Joined → False,
   PlotStyle → {Black, 0.1}, AxesStyle → Directive[Black, Thick, 14],
   PlotMarkers → {marker1F, 0.03}, PlotRange → All];
Show[{gph9F, gph10F}]

gph11F = Plot[sol2[bestk12, bestkm12][t],
   {t, startTime, endTime}, PlotStyle → {Black, 0.1},
   AxesStyle → Directive[Black, Thick, 14], PlotRange → All];
gph12F = ListPlot[{inside}, Joined → False, PlotStyle → {Black, 0.1},
   AxesStyle → Directive[Black, Thick, 14],
   PlotMarkers → {marker1F, 0.03}, PlotRange → All];
Show[{gph12F, gph11F}]
```

Out[245]=

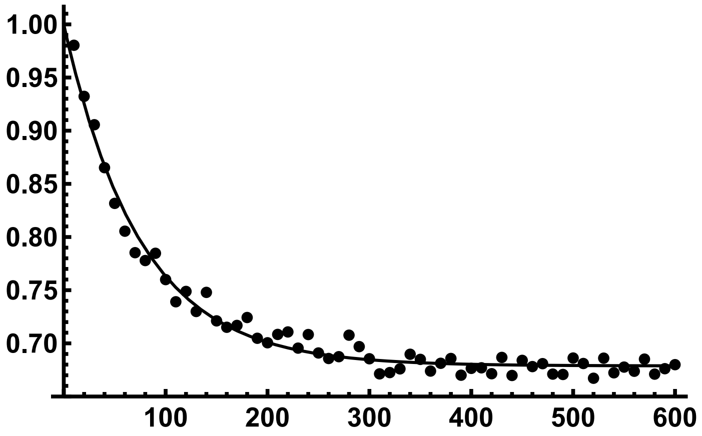

Out[248]=

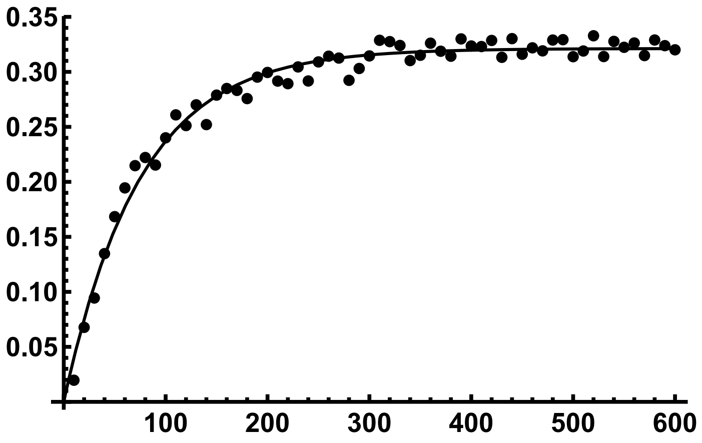

Membrane potential...assuming volume ratio outside:inside = 68:32...PWK 26/7/19

```
rR = 8.314;
tT = 310.16;
fF = 96.5 × 10^3;
ratioConcsOutOverIn =
  (sol1[bestk12, bestkm12][600] / 68) / (sol2[bestk12, bestkm12][600] / 32)
delPsi = (rR tT / fF) Log[ratioConcsOutOverIn] (* Membrane potential in volts *)
```

Out[252]= 0.994872

Out[253]= -0.000137392

$^{133}Cs^+$-dDNP uptake into RBCs...1st experiment on Tuesday June 25, 2019...PWK

Shift t=0 point for the inside peak to 0.00

```
In[33]:= dataDir = SetDirectory["/Users/philipwk77/Desktop/"];

data1 = Drop[Import["20190625_CsCl_40mM_RBC_1.xlsx"][[1]], 2];
firstScale = Max[Drop[Drop[Transpose[data1][[2]], 0], -75]]
transposeFirstData1 = Drop[Drop[Transpose[data1][[2]], 0], -75] / firstScale;
Length@transposeFirstData1
secondScale = firstScale;
transposeSecondData1 =
 ReplacePart[Drop[Drop[Transpose[data1][[3]], 0], -75] / secondScale, 1 → 0.0]
timedata1 = Range[0, Length[transposeFirstData1] - 1];
fullFirstData1 = Transpose@Append[{timedata1}, transposeFirstData1];
fullSecondData1 = Transpose@Append[{timedata1}, transposeSecondData1];

... SetDirectory: Cannot set current directory to /Users/philipwk77/Desktop/.

Out[35]= 5624.66

Out[37]= 41

Out[39]= {0., 0.000644305, 0.000948679, 0.00121056, 0.00113127, 0.00124594, 0.00108824,
 0.000922722, 0.000895165, 0.000802004, 0.000762357, 0.000756846, 0.000590436,
 0.000501008, 0.000438782, 0.000444471, 0.000412469, 0.000340998, 0.000266149,
 0.000111651, 0.000121252, 0.000157698, 0.000042847, 0.00010294, 0.0000257793,
 0.000160721, 0.00010934, 0.0000519142, 0.0000568923, 0.0000140453, 0.0000263127,
 0.0000558256, 0.0000590258, 3.20019 × 10^-6, 5.15587 × 10^-6, 0.0000368022,
 0.0000231125, 0.0000161788, 0.0000128008, 5.15587 × 10^-6, 3.91135 × 10^-6}
```

In[43]:=
```
marker1 = Graphics[{Blue, Disk[]}];
marker2 = Graphics[{Red, Disk[]}];
gph0 = ListPlot[fullFirstData1, Joined → True,
  PlotStyle → {Black, 0.1}, AxesStyle → Directive[Black, Thick, 14],
  PlotMarkers → {marker1, 0.04}, PlotRange → All]
gph1 = ListPlot[fullSecondData1, Joined → True,
  PlotStyle → {Black, 0.1}, AxesStyle → Directive[Black, Thick, 14],
  PlotMarkers → {marker2, 0.04}, PlotRange → All]
```

Out[45]=

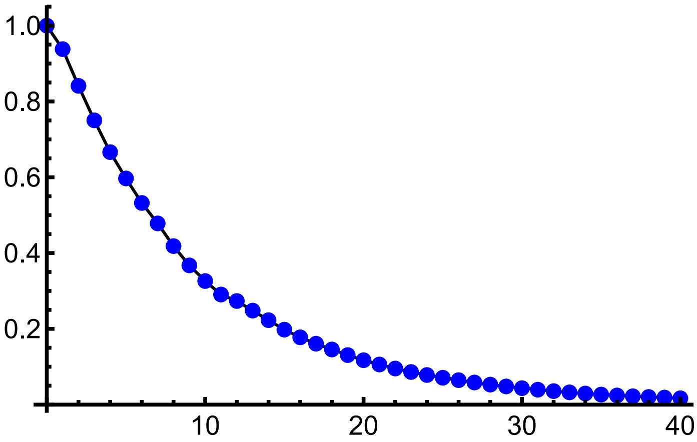

Out[46]=

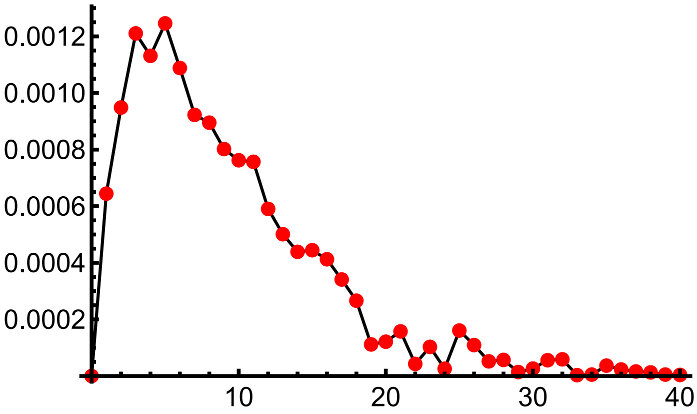

In[•]:=

Set up the solutions of the differential equations for the transport/metabolism system

In[47]:=

```
ClearAll[sol, T1o, T1i, km1, k1, co, ci];

startTime = 0;
endTime = 40.0;
co0 = 1.0;
ci0 = 0.0;
T1o = 10.0;
T1i = 2.0;
k1 = 0.0009;
km1 = 0.000;

sol = NDSolve[{co'[t] == - co[t]/T1o + km1 ci[t] - k1 co[t], co[0] == co0,
   ci'[t] == - ci[t]/T1i - km1 ci[t] + k1 co[t], ci[0] == ci0
  },
  {co, ci},
  {t, startTime, endTime}]
```

Out[56]=

```
{{co → InterpolatingFunction[ Domain: {{0., 40.}} Output: scalar ],
  ci → InterpolatingFunction[ Domain: {{0., 40.}} Output: scalar ]}}
```

In[57]:=
```
gph2 = Plot[co[t] /. sol, {t, 0, 40.0}]
gph3 = Plot[ci[t] /. sol, {t, 0, 40.0}]
```

Out[57]=

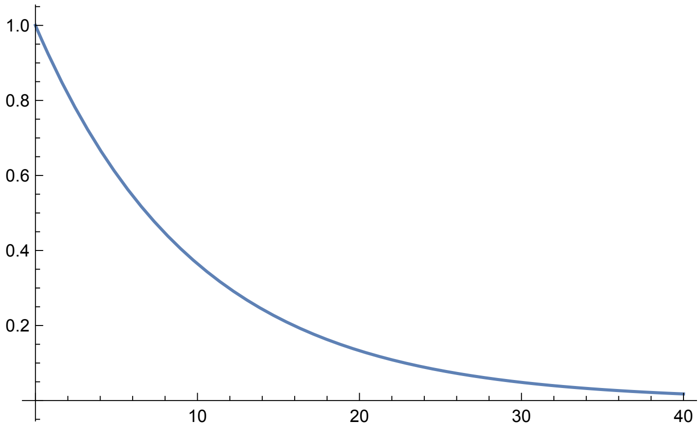

Out[58]=

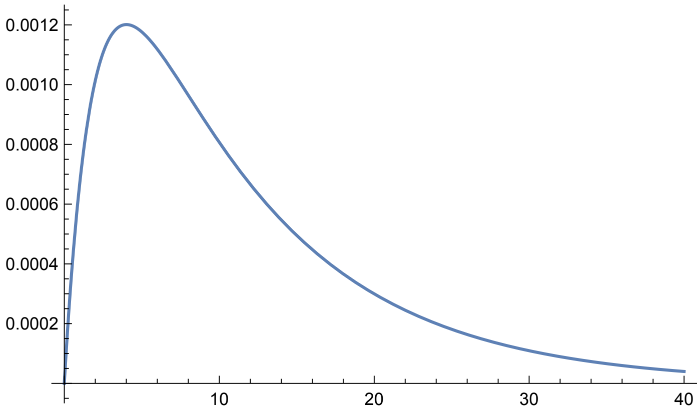

In[59]:=
```
Show[{gph0, gph2}]
Show[{gph1, gph3}]
```

Out[59]=

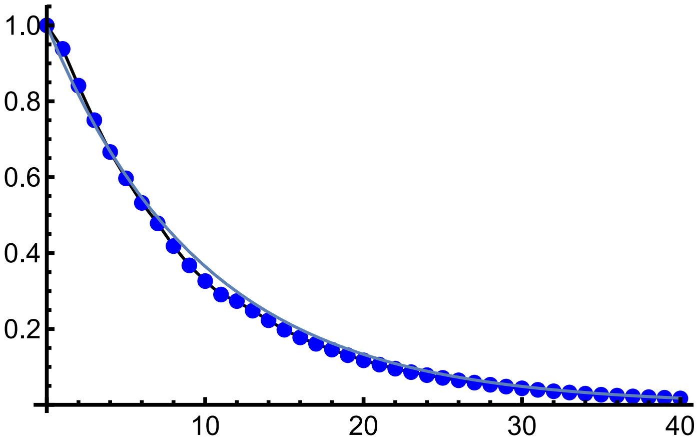

Out[60]=

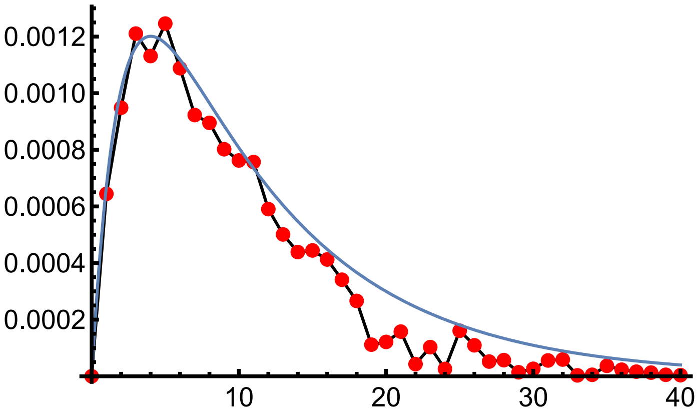

In[•]:=

The transport/metabolic model

In[61]:=
```
ClearAll[metabModel, T1o, T1i, k1, km1, co0, ci0, eq1,
  eq2, eq3, eq4, t, i, tStart, tEnd, alfa, endTime, soln, out];
out = {};
metabModel[vector_] :=
 (metabModel[vector] =
   Module[{T1o, T1i, k1, km1, co0, ci0, alfa,
     eq1, eq2, eq3, eq4, t, i, tStart, tEnd, endTime, soln, out},

    T1o = vector[[1]];
    T1i = vector[[2]];
    k1 = vector[[3]];
    km1 = vector[[4]];
    co0 = vector[[5]];
    ci0 = vector[[6]];
    alfa = vector[[7]] π / 180.0;
    (* alfa is given as degrees in the input vector...
     but it needs to be in radians for the trigonometric
     functions in Mathematica hence the conversion here *)
```

```
    endTime = vector[[8]];

    out = {};
    out = AppendTo[out, {0.0, co0, ci0}];

    (* Reaction scheme has two time-dependent variables ...
     hence two differential equations *)

                          co[t]
    eq1 := co'[t] == - ------- + km1 * ci[t] - k1 * co[t];
                          T1o
    eq2 := co[tStart] == co0;
                          ci[t]
    eq3 := ci'[t] == - ------- - km1 * ci[t] + k1 * co[t];
                          T1i
    eq4 := ci[tStart] == ci0;

    (* Loop to numerically simulate the whole
      timecourse taking into account the sampling RF-pulses *)

    For[i = 1, i ≤ endTime, i++,

     (* Define the time domain *)

     δt = 1.0;
     (* Note the specification here of the sampling interval of 1.0 s *)
     tStart = (i - 1) δt;
     tEnd = i δt;

     (* Numerically solve the differential equations *)

     soln = NDSolve[{eq1, eq2, eq3, eq4},
       {co, ci},
       {t, tStart, tEnd}];

     out = AppendTo[out, {tEnd, co[tEnd] /. soln[[1]], ci[tEnd] /. soln[[1]]}];

     (* Create the new initial conditions *)

     co0 = Cos[alfa] (co[tEnd] /. soln[[1]]);
     ci0 = Cos[alfa] (ci[tEnd] /. soln[[1]]);
    ];

    Return[out];
]);
```

In[64]:=
```
(* Some starting estimates *)

ClearAll[T1oGuess, T1iGuess, k1Guess, km1Guess, co0, ci0, alfa, endTime]

T1oGuess = 10.0;
T1iGuess = 2.0;
k1Guess = 0.0009;
km1Guess = 0.000;
co0 = 1.0;
ci0 = 0.0;
alfaGuess = 10.0;
endTime = 40.0;

cst0 = {T1oGuess, T1iGuess, k1Guess, km1Guess, co0, ci0, alfaGuess, endTime}
```

Out[73]=
```
{10., 2., 0.0009, 0., 1., 0., 10., 40.}
```

Quick test of the metabModel function

In[74]:=

```
Clear[simuln];

simuln = Transpose@metabModel[cst0];

coForPlot = {simuln[[1]], simuln[[2]]} // Transpose;
ciForPlot = {simuln[[1]], simuln[[3]]} // Transpose;

gph4 = ListPlot[coForPlot, Joined → True, PlotRange → All]
gph5 = ListPlot[ciForPlot, Joined → True, PlotRange → All]
Show[{gph2, gph4}]
```

Out[78]=

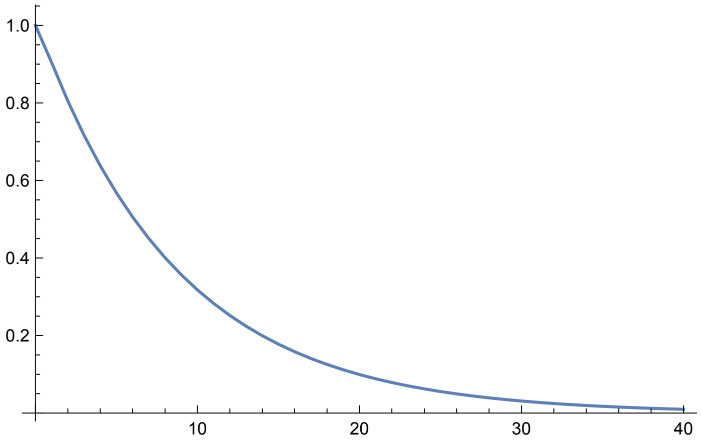

Out[79]=

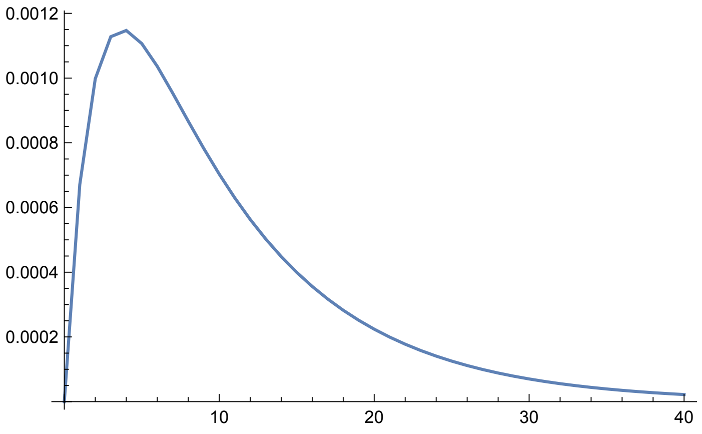

Out[80]=

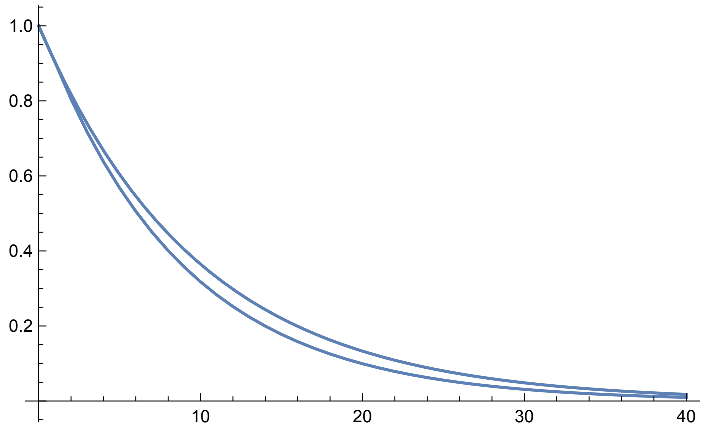

In[81]:=
Fitting

Out[81]= Fitting

In[82]:=
```
(* Calculate the least squares difference *)
lsq[vector_] := {
simuln = Transpose@metabModel[vector];
timeC = simuln[[1]];
simco = simuln[[2]];
simci = simuln[[3]];

dataco = fullFirstData1[[All, 2]];
    dataci = fullSecondData1[[All, 2]];

lsqco = (dataco - simco)^2;
lsqci = (dataci - simci)^2;

Total[lsqco + 1000 * lsqci] * 10^-6} ; (* 1000 =
 "PWKfactor" to add weight to the signal from inside the RBCs...PWK 10/6/19 *)
```

In[83]:=
```
lsq[cst0]
```

Out[83]= $\{1.4459 \times 10^{-8}\}$

In[84]:=
```
σ = 1 × 10^-5;
nit = 10 000;
likelihood[vector_] := Exp[-lsq[vector] / (2 σ^2)];
```

In[87]:=
```
likelihood[cst0]
```

Out[87]= $\{4.00656 \times 10^{-32}\}$

In[88]:=
```
(* Increment the parameters used in the MCMC algorithm *)

dT1o = 1/50 T1oGuess;
dT1i = 1/50 T1iGuess;
dk1 = 1/50 k1Guess;
dkm1 = 0.0 * 1/50 km1Guess;
dco0 = 0.0;
dci0 = 0.0;
dalfa = 1/50 alfaGuess;
dendTime = 0.0;

stepVec = {dT1o, dT1i, dk1, dkm1, dco0, dci0, dalfa, dendTime};

step[cst_] := {
cst + stepVec * RandomReal[NormalDistribution[0, 1], Length[stepVec]]}[[1]];
```

In[98]:=
```
step1 = step[cst0]
```

Out[98]=
```
{9.85642, 1.93716, 0.000852957, 0., 1., 0., 9.7778, 40.}
```

In[99]:=
```
takestep[cst_] := {
   newcst = Abs@step[cst];
   outcst = 0;
   α = (likelihood@newcst / likelihood@cst) // Last;
If[RandomReal[] < α, outcst = newcst, outcst = cst];
   outcst} // Last
```

In[100]:=
```
Print["Started at: ", DateList[][[4]], "h ",
  DateList[][[5]], "min ", IntegerPart[DateList[][[6]]], "s"];

Timing[list = NestList[takestep, cst0, nit]];

Print["Finished at: ", DateList[][[4]], "h ",
  DateList[][[5]], "min ", IntegerPart[DateList[][[6]]], "s"];
```

```
Started at: 13h 25min 57s
Finished at: 13h 30min 21s
```

In[103]:=
```
{lT1o, lT1i, lk1, lkm1, lco0, lci0, lalfa, lendTime} = Transpose@list;
Print[
  Style["Parameters have been changed (relative to 1):", Plain, Black, 14]];
accept = 1 - Count[Differences[lT1o], 0.] / nit // N
```

Parameters have been changed (relative to 1):

Out[105]= 0.4861

In[106]:=
```
(* Take only the last 70% of the chain values *)
take = Round[0.7 * nit];
chain[l_] := {
a = Mean[Take[l, -take]];
s = StandardDeviation[Take[l, -take]];
{a, s}
}[[1]]
```

In[108]:= `chain[list]`

Out[108]= {{14.1626, 1.66771, 0.00110685, 0., 1., 0., 15.6712, 40.},
{1.56394, 0.397448, 0.000227716, 0., 0., 0., 1.61876, 0.}}

Chain output

In[109]:=
```
chainout = chain[list];
averages = chainout[[1]]
stds = chainout[[2]]
```

Out[110]= {14.1626, 1.66771, 0.00110685, 0., 1., 0., 15.6712, 40.}

Out[111]= {1.56394, 0.397448, 0.000227716, 0., 0., 0., 1.61876, 0.}

In[112]:=
```
(* T1o *)
Print[Style["T1 Outside", Bold, Black, 14]];
ListLinePlot@lT1o
g[1] = Histogram@lT1o
averages[[1]]
stds[[1]]

(* T1i *)
Print[Style["T1 Inside", Bold, Black, 14]];
ListLinePlot@lT1i
g[2] = Histogram@lT1i
averages[[2]]
stds[[2]]

(* k1 *)
Print[Style["k1", Bold, Black, 14]];
ListLinePlot@lk1
g[3] = Histogram@lk1
averages[[3]]
stds[[3]]

(* k-1 *)
(*Print[Style["k-1",Bold,Black,14]];
ListLinePlot@lkm1
g[4]=Histogram@lkm1
averages[[4]]
stds[[4]]*)

(* alfa *)
Print[Style["alfa", Bold, Black, 14]];
ListLinePlot@lalfa
g[7] = Histogram@lalfa
averages[[7]]
stds[[7]]
```

T_1 Outside

Out[113]=

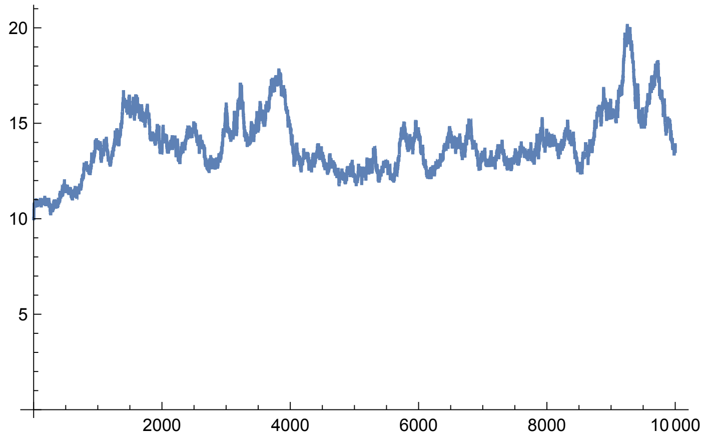

Out[114]=

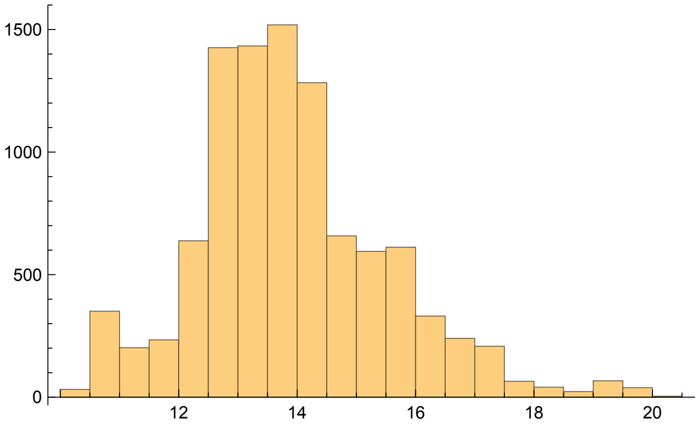

Out[115]= 14.1626

Out[116]= 1.56394

T_1 Inside

Out[118]=

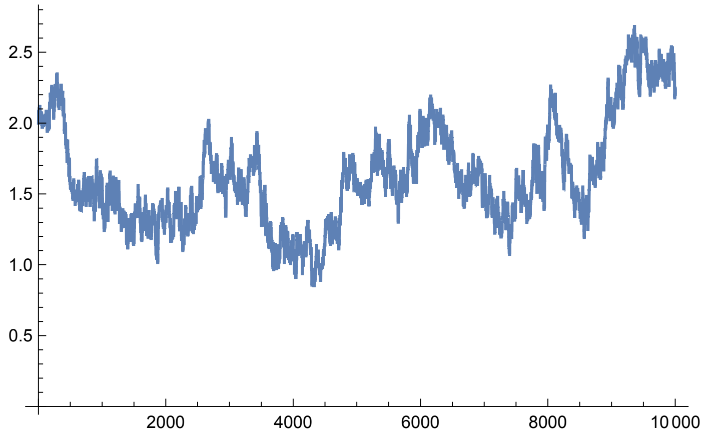

Out[119]=

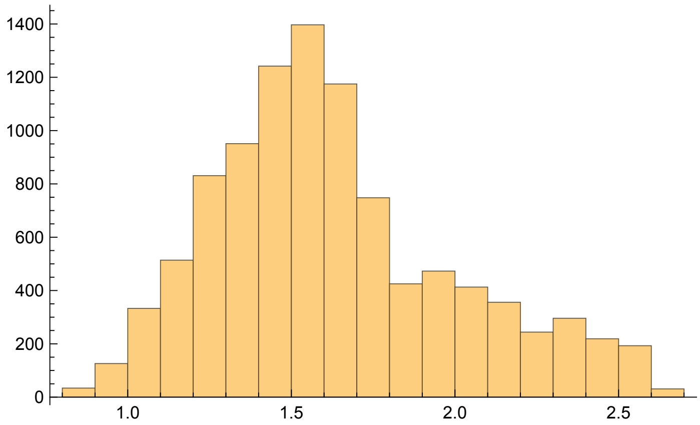

Out[120]= 1.66771

Out[121]= 0.397448

k_1

Out[123]=

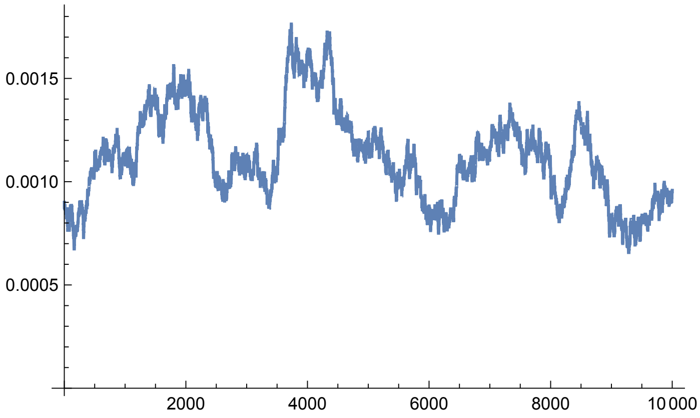

Out[124]=

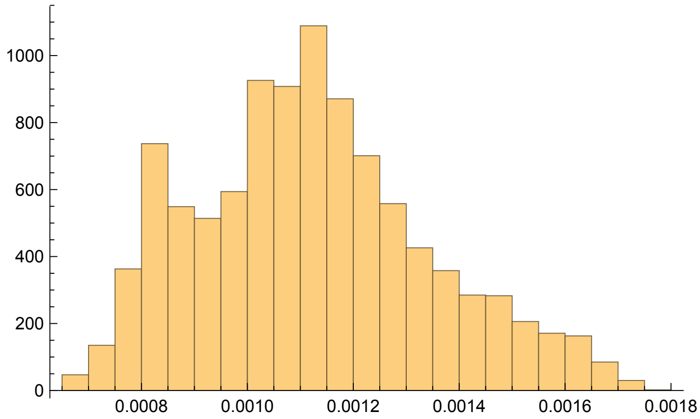

Out[125]= 0.00110685

Out[126]= 0.000227716

alfa

Out[128]=

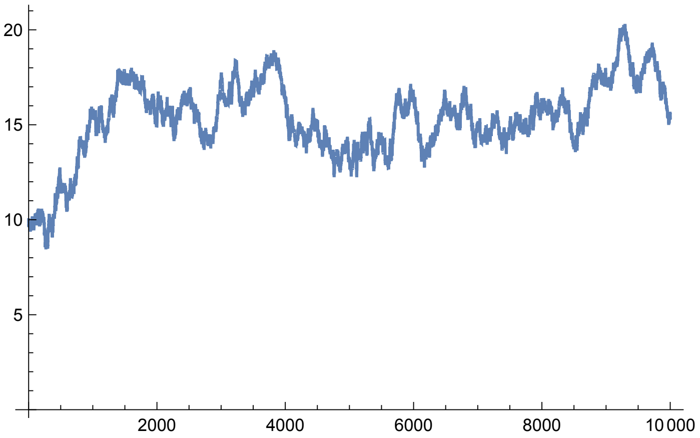

Out[129]=

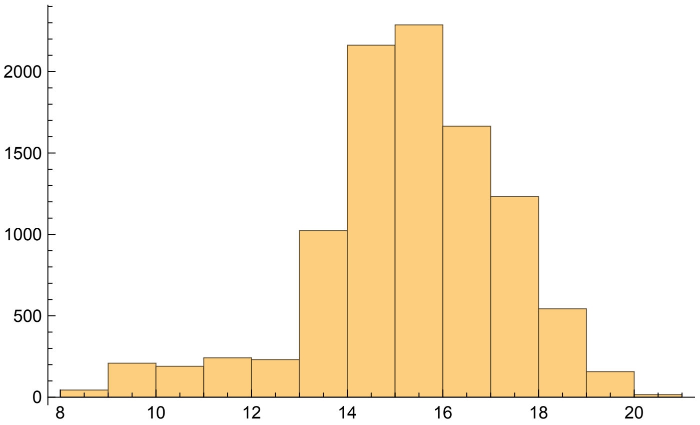

Out[130]= 15.6712

Out[131]= 1.61876

In[147]:=
```
GraphicsGrid[{{g[1], g[2]}, {g[3], g[7]}},
 Frame → All, Frame → True, Spacings → Scaled[.5]]
```

Out[147]=

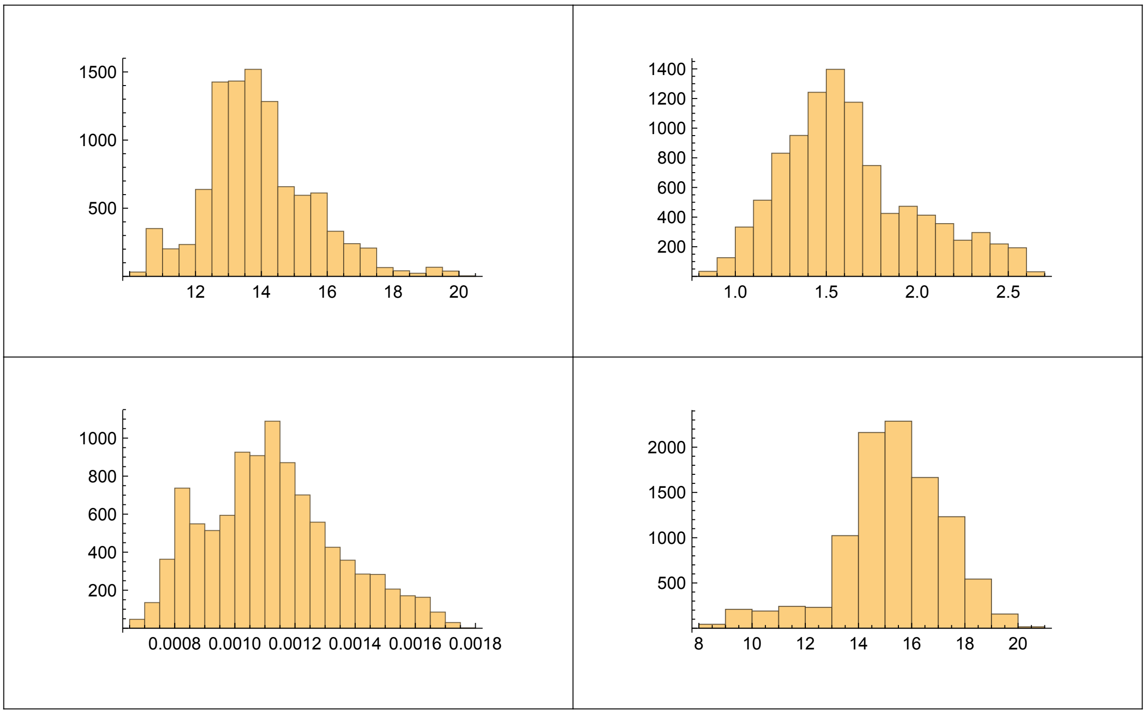

Plotting the final fit

In[133]:=
```
simuln = Transpose@metabModel[averages];
{coplot, ciplot} = Transpose@{simuln[[1]], #} & /@ {simuln[[2]], simuln[[3]]};

ListLinePlot[{coplot, fullFirstData1}, PlotRange → All]
ListLinePlot[{ciplot, fullSecondData1}, PlotRange → All]
```

Out[135]=

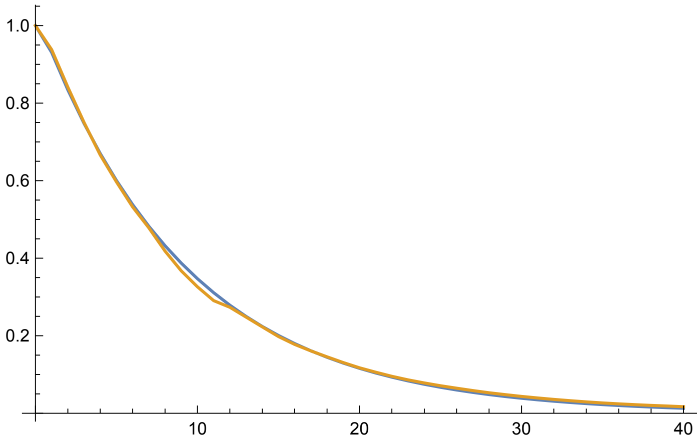

Out[136]=

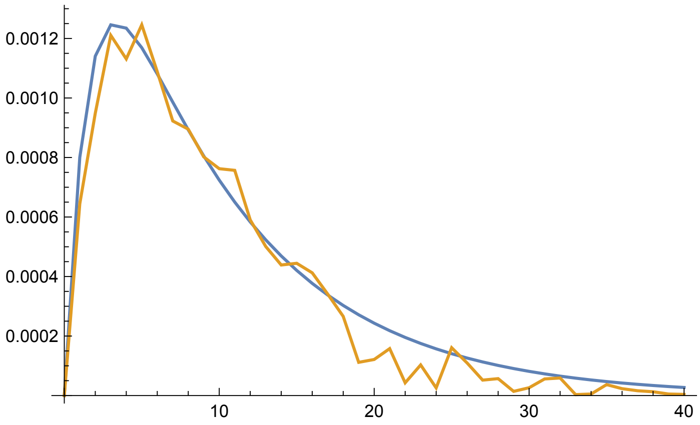

In[137]:=
```
lsq[averages]
```

Out[137]= $\{2.23263 \times 10^{-9}\}$

In[138]:=

Preparation for figures in the manuscript ... PWK Saturday 1/7/19

In[139]:=
```
coGrph = ListPlot[coplot, Joined → True,
    PlotStyle → {Black, 0.2}, AxesStyle → Directive[Thick, Black, 16]];
ciGrph = ListPlot[ciplot, Joined → True, PlotStyle → {Black, 0.2},
    AxesStyle → Directive[Thick, Black, 16]];

marker1F = Graphics[{Black, Disk[]}];
marker2F = Graphics[{Black, Disk[]}];

gph09 = ListPlot[fullFirstData1, Joined → False,
    PlotStyle → {Black, 0.1}, AxesStyle → Directive[Black, Thick, 14],
    PlotMarkers → {marker1F, 0.03}, PlotRange → All];
gph19 = ListPlot[fullSecondData1, Joined → False,
    PlotStyle → {Black, 0.1}, AxesStyle → Directive[Black, Thick, 14],
    PlotMarkers → {marker1F, 0.03}, PlotRange → All];

Show[{gph09, coGrph}]
Show[{gph19, ciGrph}]
```

Out[145]=

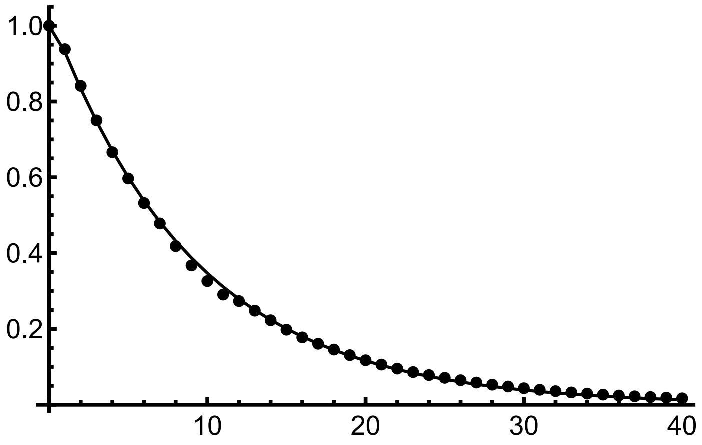

Out[146]=

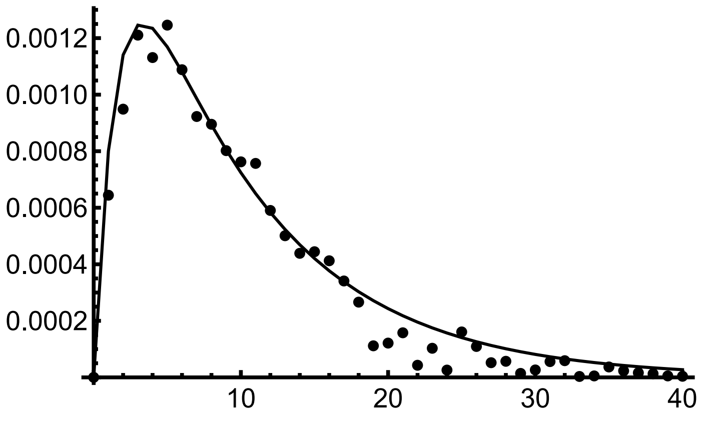

Supplement: Supplementary file 1 — Supplementary Information [file 41598_2019_56250_MOESM1_ESM.pdf]
